# What Happens in TBI? A Wide Talk on Animal Models and Future Perspective

**DOI:** 10.2174/1570159X20666220706094248

**Published:** 2023-04-12

**Authors:** Satyabrata Kundu, Shamsher Singh

**Affiliations:** 1 Department of Pharmacology, ISF College of Pharmacy, Moga, Punjab, India

**Keywords:** Traumatic brain injury, social impairment, pathophysiology, craniotomy, neuroinflammation

## Abstract

Traumatic brain injury (TBI) is a global healthcare concern and a leading cause of death. The most common causes of TBI include road accidents, sports injuries, violence in warzones, and falls. TBI induces neuronal cell death independent of age, gender, and genetic background. TBI survivor patients often experience long-term behavioral changes like cognitive and emotional changes. TBI affects social activity, reducing the quality and duration of life. Over the last 40 years, several rodent models have been developed to mimic different clinical outcomes of human TBI for a better understanding of pathophysiology and to check the efficacy of drugs used for TBI. However, promising neuroprotective approaches that have been used preclinically have been found to be less beneficial in clinical trials. So, there is an urgent need to find a suitable animal model for establishing a new therapeutic intervention useful for TBI. In this review, we have demonstrated the etiology of TBI and post-TBI social life alteration, and also discussed various preclinical TBI models of rodents, zebrafish, and drosophila.

## 1 INTRODUCTION

Traumatic brain injury is a leading cause of mortality and morbidity, and occurs when a sudden force is transmitted to the head, leading to neurological degeneration. Degenerative diseases of the nervous system are characterized by progressive loss of neurons, resulting in altered neurotransmitters levels [1]. Several neurodegenerative diseases, like Parkinson’s disease (PD), Alzheimer’s disease (AD), Autism spectrum disorder (ASD), and Huntington’s disease (HD) play a major role in neurodegeneration, and traumatic brain injury (TBI) is associated with an increased risk of all these neurodegenerative diseases [2]. Nearly 40% of injury-related deaths in the United States are caused by traumatic brain injuries. Centers for Disease Control (CDC) report showed that approximately 1.72 million people in the United States are facing TBI, and 275,000 candidates are hospitalized per year [3, 4]. Over 50,000 people die annually from these experiences, resulting in 70,000 Americans who live with long-term impairments, with 5.3 million Americans only in the U.S. with these disabilities, which have a profound emotional and socioeconomic impact on society and families [3]. There is a low life expectancy seen among survivors of severe traumatic brain injury. The survivors face prolonged care and rehabilitation, and consequently, long-term physical and mental damage affects their relationships and employment. Anestimated lifetime cost per case of severe TBI patients was reported to be US$396,331, where US$330,827 was incurred for disability and productivity lost cost [5]. TBI constitutes a heterogenous condition that affects the brain diversely. The term traumatic brain injury is also referred to as intracranial injury, which leads to mood instability, disability in movement [6], sensitivity to light with blurred vision, cognitive decline, and behavioral alterations [7]. TBI gives rise to focal injuries and diffuse axonal injuries (DAI) due to shearing, tearing, and stretching of axons. The commonest events of TBI include falls, vehicle collisions, and sports injuries like in boxing, football, and hockey. Most TBI incidences are observed in a very young age group (0-4 years) as well as in adolescents and young adults (15-24 years). There is another peak of traumatic brain injury observed after the age of 65 years, with higher morbidity, mortality, and slow recovery [8]. In severe TBI, injuries lead to disruption of the blood-brain barrier (BBB), intracranial hematoma, formation of brain edema, free radical generation, depolarization, excitotoxicity, and imbalance in calcium homeostasis. Abnormal increase of reactive oxygen species and increase in proinflammatory cytokines production play a key role in primary cell death [9]. Secondary neuronal degeneration (SND) is considered one of the most significant events associated with TBI. SND mainly initiates various downstream cascades of destructive events that may affect the cells primarily affected by initial wounds [4]. However, secondary injury in TBI occurs after hours to days with cell structural changes and alteration in various neurotransmitters, like dopamine (DA), serotonin, GABA, and glutamate, resulting in neuronal cell death [10]. The degree of secondary injury damages is proportionally accompanied by the extension of initial injuries. So, the more lasting the primary injuries, there will be a greater release of mediators of SND [4].

Pathophysiology of SND is related to the participation of various key factors following TBI. Among these, disbalance in neurotransmitters, oxidative stress, and the inflammatory response seem to be involved widely in tissue degeneration.

Numerous animal models have been performed to replicate the human TBI consequences. The speed of impact between human head and object is a crucial factor influencing outcomes of brain injury [11]. The impact acceleration model of TBI was developed to produce diffused axonal injuries in rodents without the presence of fractured skull and parenchymal focal lesions. This model imitates the pathophysiology of human diffuse axonal injury caused by sudden forces (vehicle accidents, falls) to the brain. The impact acceleration model can help in producing axonal injury in the absence of a focal contusion [12].

## 2 ETIOLOGY OF TRAUMATIC BRAIN INJURY

### 2.1 TBI-mediated Disbalance in Excitatory and Inhibitory Neurotransmitter

Glutamate and γ-aminobutyric acid (GABA) are the primary excitatory and inhibitory neurotransmitters in the brain, respectively. For normal neurological function, the balance between glutamate and GABA is essential; but in TBI, glutaminergic activity is raised twofold. On one side, the acute post-traumatic release of glutamate is linked with neuronal insult, cell death, and loss of neuronal activity, whereas, on the other side, slow glutamate signaling results in motor and cognitive impairment [13]. In the cerebral cortex, glutaminergic neurons comprise 80% of the total neuronal population. Post-traumatic or ischemic injury in the brain initiates a high concentration of extracellular glutamate, resulting in inotropic glutamatergic receptors activation and, consequently, alteration in Na^+^/K^+^ pump with sodium and chloride ions influx, resulting in increased water uptake, which leads to a high volume of cell body [14]. Recently, in TBI research, α1, γ2, α4 and δ1 subunits of GABA-A have occupied most attention. These subunits contribute to GABA interneuron's ability to modulate neuronal signaling *via* phasic (α1, γ2) and tonic (α4, δ1) inhibition [13]. Phasic inhibition decreases the hyperexcitability of the post-synaptic cells, whereas tonic inhibition maintains the amount and duration of post-synaptic depolarization [15].

In TBI research, NR1, NR2A and NR2B subunits of N-methyl-D-aspartate receptor (NMDAR) have gained the most attention. The glycine binds with the NR1 subunit, which is responsible for the deactivation of the receptor [16]. NR2A containing NMDA receptors are localized with NR1 subunit, primarily at the synapse, and stimulation of these receptors reinforces synapses and encourages several signals, including phosphorylated ERK, phosphorylated CREB, and brain-derived neurotrophic factor [17]. Whereas, NR2B containing NMDARs are restricted extrasynaptically, and activation of this receptor leads to cell death. NR2B activation results in prolonged calcium influx, damage to mitochondria as well as increased activation of caspases, being further responsible for neuronal cell damage [18].

TBI also alters the regulation of α-amino-3-hydroxy-5-methyl-4-isoxazolepropionic acid receptor (AMPAR) expression, which further leads to excitotoxic injury. After TBI, GluR1 receptor upregulation increases calcium influx and calcium/calmodulin-dependent protein kinases (CaMKs) activation. CaMKs activation is associated predominantly with normal memory function, and its dysregulation or inactivation leads to impaired memory function [19]. Studies established that an *in vitro* and *in vivo* TBI model showed a decrease in NMDAR-mediated GluR2 subunit expression [20]. Reduced GluR2 leads to an increase in intracellular calcium flux through AMPARs and NMDARs, which is responsible for post-TBI vulnerability and neuronal cell death as well [21].

### 2.2 TBI-mediated Apoptotic Signaling Alteration

Programmed cell death or apoptosis of neurons and glial cells contribute to the overall pathology of TBI in both humans and animals. Reported data suggest that in the post-traumatic brain, a low level of intracellular calcium leads to apoptosis, whereas a high level of calcium can cause necrosis. TBI brain faces complicated consequences, including disbalance in anti-apoptotic and pro-apoptotic cell death signaling pathways. For example, the death-inducing Bcl-2 family (Bax, Bad. Bid) shows dynamic equilibrium with survival-promoting congeners (Bcl-2, Bcl-x_L_) [22]. In the acute post-traumatic period, apoptotic oligodendrocytes and astrocytes have been identified within contusions. Post-TBI neuronal apoptosis is well accepted, but the signaling mechanism is still doubtful [23].

#### 2.2.1 Extrinsic Signaling Pathways

Tumor necrosis factor (TNF) and extracellular ligands (Fas-Fas ligand) activate apoptotic extrinsic signaling pathways. On the cell surface of the death receptor, both TNF and Fas-Fas ligand are attached and exhibit activation of the death-inducing signaling complex (DISC). The activated macrophage releases various cytokines, such as TNF-α, a major mediator of apoptosis. Activation of initiator caspases (caspase 8 and caspase 10) is stimulated by the binding of TNF-α on its receptors (TNFR1 and TNFR2). A type II membrane protein (Fas ligand, CD95L) is responsible for apoptotic cell death mediated by the activation of caspases [24]. In TBI, activated macrophages produce the major cytokine TNF-α, and its activation further upregulates caspase-dependent apoptogenic signaling pathways [25].

#### 2.2.2 Intrinsic Signaling Pathways

The apoptotic intrinsic pathways are initiated by stress on cellular organelles, including mitochondria and the endoplasmic reticulum (ER). Stress on ER activates the PERK-eIF2α pathway that improves cell function, whereas unresolve/intense ER stress activates C/EBP homologous protein (CHOP), leading to cell death. TBI alters intrinsic signaling pathways by various mechanisms, which are discussed below.

##### 2.2.2.1 Calcium Signaling Pathways Alteration

In the normal cellular and physiological processes, calcium acts as a transduction molecule for the activation of various enzymes (cyclic nucleotide phosphodiesterase (PDE), adenylyl cyclase, and nitric oxide synthase). Various apoptotic signaling pathways are activated in response to calcium elevation (1000 nM) from its normal level (100 nM) [26].

###### 2.2.2.1.1 Alteration of Calcium Dependent-phospholipase C Pathways Post-TBI

TBI may activate apoptotic signaling by stimulating phospholipase C (PLC) pathways. PLC isozymes hydrolyze phosphatidylinositol 4, 5 bisphosphates (PIP2) into the secondary messengers to form inositol triphosphate (IP3) and diacylglycerol (DAG). The formation of diacylglycerol (DAG) activates several types of protein kinases, which finally induce apoptosis. When IP3 binds to its receptor (IP3R), the release of calcium activates calcium-calmodulin-dependent and caspase-dependent apoptotic pathways. Excessive release of calcium-mediated apoptosis is shown in Fig. (**1**) [27].

The post-traumatic brain experiences a hike in intracranial pressure, resulting in the breakdown of micro-vessels (primarily, ruptured endothelial cells also include pericytes, pre capillary arteriolar smooth muscle cells, and astrocytes). Ruptured micro-vessels release cytotoxic levels of iron into the brain parenchyma. The Ca^2+^-dependent mechanism is promoted by iron, which can either stimulate cell survival or cell death based on the severity and duration of iron exposure [28].

###### 2.2.2.1.2 Induction of Calcium-dependent Endoplasmic Reticulum (ER) Stress Pathway Post-TBI

Endoplasmic reticulum (ER) stress has a role in the activation of the apoptotic signaling pathway. ER plays a major role in the maintenance of intracellular calcium homeostasis. After TBI, increased release of calcium leads to calpain activation, followed by activation of caspase-12, caspase-9, and caspase-3. The activated caspase-3 leads to the breakdown of DNA, which is a prominent hallmark of apoptosis [29].

### 2.3 TBI-mediated Activation of Oxidative Stress Signaling Pathways

Oxidative stress is caused by the imbalance between antioxidant enzymes and reactive oxygen species (ROS), which initiates the production of lipid peroxidase (LPO) in the cellular, mitochondrial and nuclear membranes. The production of free oxygen radicals, superoxide, nitric oxide and peroxy-nitrite potentiates the release of glutamate, followed by impairment of energy metabolism [30]. Endogenous antioxidant systems, including glutathione peroxide, superoxide dismutase, and uric acid, neutralize those reactive oxygen species, thus preventing ROS from binding to several macromolecules, like DNA, RNA, or proteins. Excessive production of ROS interacts with the endogenous antioxidant system and results in inhibition of the mitochondrial electron transport chain (ETC) [31].

Nitric oxide (NO) is a lipid and water-soluble free radical that acts as an autocrine signaling molecule as well as a paracrine signaling molecule. NO is produced from the amino acid L-arginine by the nitric oxide synthase (NOS) protein family. NOS family catalyzes the conversion of L-arginine first to N-hydroxyl-arginine, and then to L-citrulline and NO•. NOS activation requires the binding of calmodulin (CaM). Numerous members of the NOS family are known to regulate various cellular functions [32]. In the central nervous system, the production of NO is associated with cognitive function, induction, and maintenance of synaptic plasticity for controlling body temperature, appetite, and neurosecretion [33]. Garthwaite *et al.* reported that cerebellar NMDA receptors stimulation by glutamate is responsible for diffusible molecule release with a strong similarity to endothelial-derived relaxation factor (EDRF) [34]. In the telencephalon and the cerebellum, NO shows synaptic plasticity regulation involved in long-term potentiation and long-term depression of synaptic transmission. Nitrosative stress involved in neurodegenerative disorders is no longer a matter of concern. In these disorders, excess NO is produced by inducible nitric oxide synthase (iNOS) induction owing to the pro-inflammatory response. Furthermore, under pathological conditions, NO is much more harmful, as it is involved in the production of ROS (superoxide anions and peroxynitrite). The interaction between peroxynitrite (ONOO^-^) and cellular elements leads to protein nitration, resulting in cellular elements damage. It has been proposed that S-nitrosylation (S-NO) of certain substrates (parkin90,91, GAPDH92, matrix metalloproteinase 9) can show neurotoxic effects [35].

Various animal models of TBI have discussed the role of ROS associated changes in mitochondrial membrane potential that also upregulates caspase pathways (dependent and independent). In the caspase-independent pathway, degradation of chromosomal DNA is mediated by the release of apoptosis-inducing factor (AIF) or Endo G from mitochondria intermembrane [36]. An increased level of reactive oxygen species in mitochondria can trigger the complex formation of cytochrome c (Cyt c) and apoptotic protease activating factor 1 (Apaf-1), and this complex further activates caspase-9. Activated caspase-9 subsequently activates caspase 3, and activated caspase-3 leads to DNA fragmentation or cytoskeleton protein degradation [37].

Fas and Fas ligand (FasL) are members of the tumor necrosis factor. The post-traumatic brain stimulates the ligation of FasL to the Fas death receptor (FasR), which initiates the recruitment of the death domain protein (DD) that binds to procaspase 8. The activated caspase-8 enzyme further activates caspase 3. Activation of caspase 3 leads to cleavage of PARP (DNA repair enzyme). This PARP causes damage to neurons by DNA lesions or fragmentation [38]. Activation of caspase signaling after TBI is shown in Fig. (**1**).

### 2.4 TBI-mediated Disbalance in p53 Signaling Pathways

The introduction of cellular stress shows different major roles of the transcription factor (p53). Alteration in p53 activation results in the initiation of apoptotic signaling pathways after brain injuries.

In TBI, damaged DNA releases transcription factor (p53), which leads to dysregulation in the level of Bcl-2, Bax, and Bad. Bax induces apoptosis, whereas Bcl-2 prevents the apoptotic signaling process [39]. Alteration of Bcl-2 and Bax leads to cytochrome C and Apaf-1 release and forms apoptosome. In the mitochondria, the level of pro-apoptotic proteins (Bad, Bax) is increased after TBI [40]. Apoptosis induction post-TBI with the activation of p53 signaling is shown in Fig. (**1**).

### 2.5 TBI-mediated Activation of Inflammatory Signaling Pathways

The role of glial cells is to sustain and maintain neuronal function, and this is also true for microglia. Microglia, as a primary mediator of the immune system of CNS, is an integral part of the various inflammatory responses [41]. Till now, the role of microglia in the injured CNS is still under research. In the brain of healthy people, microglia are in a resting phase and have numerous dendritic morphologies. Microglia induce substantial changes to cell morphology in response to injury to the brain. On activation, microglia contract and undergo morphological changes resembling amoeboid cells found in the blood. They then proliferate and migrate toward the site of injury. However, short-term activation of microglia may have favorable effects. Under the steady-state, by removing damaged or unnecessary neurons and synapses, microglia show CNS homeostasis regulation. Microglia and CNS-infiltrating macrophages play an important role in defense mechanisms against pathogens by the regulation of innate immunity as well as various immune responses [42]. The category of M1 and M2 microglia is more apparent in various neurodegenerative disorders. M1 microglia is considered to be more inflammatory than M2 microglia. M2 microglia are characterized by increased anti-inflammatory cytokine production and reduction in NO production. M2 microglia also present specific antigens, including mannose receptor, arginase 1 in chitinase 3-like 3 and inflammatory zone 1. They are also involved in healing wounds, repairing tissue, and remodeling of extracellular matrix. Moreover, microglia exposed to IL-4 have a role in the reduction of proinflammatory cytokine production and promoting growth factor production [42, 43].

Post-TBI, damaged cell releases several endogenous factors, including RNA, DNA, heat shock proteins (HSP), and high mobility group box 1(HMGB1). The endogenous factors bind to Toll-like receptors (TLRs), which activate myeloid differentiation primary response 88 (MYD88) dependent pathways. MYD88-dependent pathway is categorized into NFκB and mitogen-activated protein kinase (MAPK). Both these pathways start to transcript various downstream genes, followed by the release of pro-inflammatory cytokines [44]. These downstream genes release various inflammatory cytokines, as shown in Fig. (**2**).

### 2.6 TBI-mediated Disbalance in mTOR Pathways

The mammalian target of rapamycin (mTOR) regulates eukaryotic cell growth and proliferation by promoting anabolic processes (biosynthesis of proteins and lipids) and limiting catabolic processes (autophagy) [45]. After TBI, several growth factors bind to growth hormone receptors, which further activate MAPK and PI3K. MAPK regulates downstream signaling pathways (increased level of MEK activates ERK). After the brain injury, the level of ERK is also increased and initiates apoptosis. Whereas decreased PI3K level initiates PDK1 (Phosphoinositide-dependent kinase-1) activation. Also, PI3K activates the protein kinase B, and the decreased level of protein kinase B results in mTOR activation [46]. Phosphorylated mTOR positively regulates ULK-1, which primarily inhibits different adaptor proteins, including Becclin1, p62, and LC3II. Whereas AMPK negatively regulates ULK-1. When the brain injury occurs, activation of AMPK starts regulating ULK-1 positively and suppresses ULK-1 regulation of mTOR (negative regulation). AMPK-associated ULK-1 activation forms the ULK-1 complex, which further promotes the biogenesis of the macroautophagic process [47]. Upon PI3K activation, Akt is activated directly, followed by indirect activation of PDK1. mTORC2 is indirectly stimulated by PDK1. Activated Akt inhibits the expression of both TSC1 and TSC2 complex protein (helps in cell growth and proliferation), which further inhibits Rheb protein (regulates cell cycle). Rheb inhibition suppresses the activation of mTORC1. This inhibition indirectly inhibits autophagy and exerts apoptosis through altering S6K and 4EBP1 proteins level [48].

The Ras-Raf-MEK-ERK pathway is mainly associated with cell proliferation. Upon activation of ERK, it inhibits the TSC1/2 complex and similarly stimulates mTORC1 [49]. There is doubtful evidence with respect to the role of mTOR after TBI. Some studies suggest that the role of mTOR is associated with cell death and neuronal protection, whereas others report post-traumatic brain injury and that mTOR signaling may promote neuroregeneration. The mTOR pathway is activated after TBI, as shown in Fig. (**3**).

## 3 IMPACT ON SOCIAL LIFE POST-TBI

### 3.1 Social Nature Impairment

The social life of TBI survivor patients is altered (frustration, increased anger or aggressiveness, difficulties in self-controlling), which continues for a longer period. The post-traumatic outcome assessment is complex and specific outcomes of brain damage are sometimes difficult to report. Severe road accidents are associated with extra-cranial injuries with major disabilities, ocular damage, amputations, and pelvic fractures that mostly affect the quality of life (QoL). However, post-traumatic patients may show a change in social outcomes in the direction of either further recovery phase or condition worsening phase. In a study, people with a closed head injury and no head injury were assessed for social interaction by a questionnaire, including the number of close friends, social discomfort, social outing, and loneliness. There was no evidence of decreased social interaction in the TBI group, but the severely injured group had shown a decreased number of friends (*p* < .04) [50]. A case report study in the USA showed that almost 73% of mildly injured patients returned to their previous job positions, while for some severely injured patients, the percentage fell to 49% [51].

### 3.2 Psychiatric Disorders

After TBI, several short-term complications may cause disbalance in the level of neurotransmitters (dopamine, serotonin, norepinephrine, and acetylcholine), resulting in psychological disturbances. In acute TBI conditions, there is an increase in dopamine levels, whereas chronic TBI conditions are associated with a decrease in dopamine levels [52]. Post-traumatic patients show neuronal hyperexcitability, which leads to the opening of calcium channels as well as dopaminergic neuron activation followed by dopamine release. Calcium channel activation enhances tyrosine hydroxylase activity and leads to the synthesis of dopamine [53]. A high level of dopamine secretions has been found to induce psychiatric disorders, including mania, depression, and dementia [54].

Serotonin reuptake transporters (SERT) mainly regulate serotonin release in the synaptic cleft and regulate synaptic activity [55]. Abe *et al.* demonstrated that in the cortical contusion area of the brain, the SERT immunoreactivity decreased after 1-14 days of injury, and at 7 days of injury, SERT mRNA and protein expression were significantly decreased [56]. These findings demonstrate that low SERT expression in TBI patients is also linked with reduced serotonin transmission. The serotonin hypothesis of depression establishes that a low level of serotonin leads to increased chances of depression [57].

Glutamate is an essential amino acid having various notable functions in the brain. Dysregulation of glutamate leads to neurotoxicity and also has a deleterious impact on neurotransmission. Various post-mortem studies show that glutamate abnormalities are linked with psychiatric disorders. For instance, in the postmortem frontal cortex of mood disorder or bipolar disorder patients, the level of increased glutamate was identified [58]. While in the hippocampus of patients with depression, altered expression of glutamate-related genes was observed [59]. Radioligand binding studies showed less glutamate attachment to kainate receptors in the region of medial temporal cortices of schizophrenics, whereas increased binding of glutamate to AMPA and NMDA receptors of frontal cortices of schizophrenics [60, 61]. In a study, bipolar disorder patients were associated with altered levels of excitatory amino acid transporters (EAATs) in the postmortem prefrontal cortex [62].

Oxidative stress has a major role in initiating psychiatric disorders. Various published data show the association of oxidative and nitrosative stress in depression pathophysiology. Elevated levels of ROS and RNS, including peroxide [63] and NO [64, 65], and dysregulation of glutathione levels in the postmortem depressed patients have been observed [66]. Some studies have also reported altered levels of neuronal energy, antioxidant enzymes, nitric oxide, lipid peroxidase, thiobarbituric acid reactive substance (TBARS), and increased NO levels in bipolar disorder patients [67, 68].

In penetrating or non-penetrating head injury, the neuropsychological symptoms vary because penetrating trauma shows psychiatric symptoms depending on the affected area (aggression and behavioral disturbances); similarly, non-penetrating trauma is associated with calcium and magnesium level dysregulation, hyper-excitotoxicity, neurotransmitter disbalance, and diffuse axonal injury. Post traumatized patients show 14-77% depression, 2-14% dysthymia, 2-17% bipolar disorder, 4-17% panic disorder, 2-15% obsessive-compulsive disorder, 3-27% posttraumatic stress disorder, and 1% schizophrenia [69]. One study reported that women are more prone to show a higher risk of developing psychiatric problems with common symptoms of headache, dizziness, lack of confidence, and lack of initiative than men among post-traumatic patients [70].

Many studies have reported an increased suicidal tendency in patients with brain injury. A study on 42 TBI survivors reported that about 10% of patients had suicidal thoughts and about 2% of the survival patients had attempted suicide after 1 year of injury [71]. One report showed the altered binding of NMDA receptors in the wider region of the brain of suicide-prone patients [72]. Many patients suffer from mania, but this probability is less compared to depression. Damage to the basal region of the right temporal lobe and right orbitofrontal cortex is related to the development of maniac symptoms in post-traumatic patients [73].

Some evidence shows that there is a high incidence of psychiatric disorders with depression and bipolar disorders having a ratio of 2.1 and 1.85, respectively. Psychiatric outcomes may be for a short period or may be persistent. But long-term psychiatric disorders may contribute greater risk to social life [74]. Moderate to severely brain-injured patients are linked with olfactory function impairment and also increased prevalence of psychiatric disorders [75].

### 3.3 Impairment of Social Relationships and Leisure Activities

Physical, emotional, and cognitive decline after brain injury prevents traumatized survivor patients from re-entry into the community. Depression, loneliness, and decreased social interaction combined with unemployment, marital problem, decrease in leisure activities, and social isolation may disrupt previous social bonding among each other [76]. One study reported that 81% of post-traumatized patients had not returned to previous leisure activities, including partying, drug and alcohol use, and several sports curricula [77]. Additionally, involvement in leisure activities could foster the development of physical activities for post-traumatic patients.

### 3.4 Sexuality

There are several controversies on whether patients show an increase, decrease, or the same sexual desire after head trauma. The traumatized brain can directly or indirectly affect sexuality or sexual function. The physical and psychological alteration may result in impairment of sexual performance. An increase in oxidative stress affects physiological properties of eNOS (eNOS uncoupling). After that, nitric oxide reacts with superoxide O_2_^–^ and forms peroxynitrite (ONOO^-^), and then eNOS produces reactive oxygen species. Uncoupling of eNOS leads to decreased NO bioavailability. A decrease in NO bioavailability is linked with the enhancement of preexisting oxidative stress. This mechanism leads to endothelial dysfunction (erectile dysfunction) [78]. However, different sexual activities, including sexual drive, erection, and ejaculation, are known to be affected [79]. One study explained that head-injured patients make frequent sexual demands, whereas another study showed a patient with head injuries to have hypersexuality along with a 3-fold increase in expected orgasms [80]. On the other side, a study on sexual activity after 1 year of TBI showed a significant decrease in sexual arousal in mild traumatized patients [81].

### 3.5 Endocrinopathies

The post-traumatized patients show hormonal imbalance due to chronic dysfunction in the pituitary axis. Post-traumatic patients show neuroendocrine dysfunction and patients with hypopituitarism may undergo anergia, osteoporosis, reduced lean body mass, decreased muscle mass, decreased energy, mood disorder, cognition impairment, insomnia, and infertility. But these symptoms are common in TBI [82], and patients with pituitary dysfunction can also have an impact on a rehabilitation course. In the study by Young *et al.*, men with hypogonadism after TBI were found to have low testosterone levels, which further shows the lower functional status and longer length of stay in rehabilitation [83].

Post-traumatic female patients experience neuroprotection and neurorecovery, which is provided by female sex hormones. In a pre-clinical rodent TBI model, estrogen and progesterone exert neuroprotection as well as neuroregeneration. The researcher evaluated that estrogen may show neuroregeneration because it has been found that estrogen receptors are somehow expressed in the cerebral affected area, and activation of these receptors leads to transcription followed by neurogenesis [84]. Post-traumatic female patients may experience menstrual dysfunction, which is an indicator of endocrine dysfunction, and has been demonstrated as worse outcome [85].

## 4 ANIMAL MODELS, PATHOGENESIS, AND UTILIZATION

Numerous animal models have been developed to mimic the various heterogeneous clinical situation of TBI. Previously, the freeze lesion model was used in cats, dogs, and macaque monkeys [86]. Also, larger animals, like pigs and sheep, have been addressed in TBI research. As larger animals show similar physiology and size, various rodent models have been established in TBI research for their low cost, small size, and perfect measurable outcomes. Previous models of TBI were associated with biochemical aspects of head injury [87], but the more recent models of TBI have targeted the complex molecular cascades caused by brain injury. Many rodent models are available, but some models are more prominent with human head injuries, including fluid percussion model (FPI), controlled cortical impact (CCI) injury, weight drop impact acceleration injury and blast injury. Currently, drosophila and zebrafish have gained more attention in TBI research because of their easy availability, short lifespan, and standardized outcome measurements.

### 4.1 Weight Drop Model

#### 4.1.1 Marmarou Weight Drop Model

**Fig. (4):**
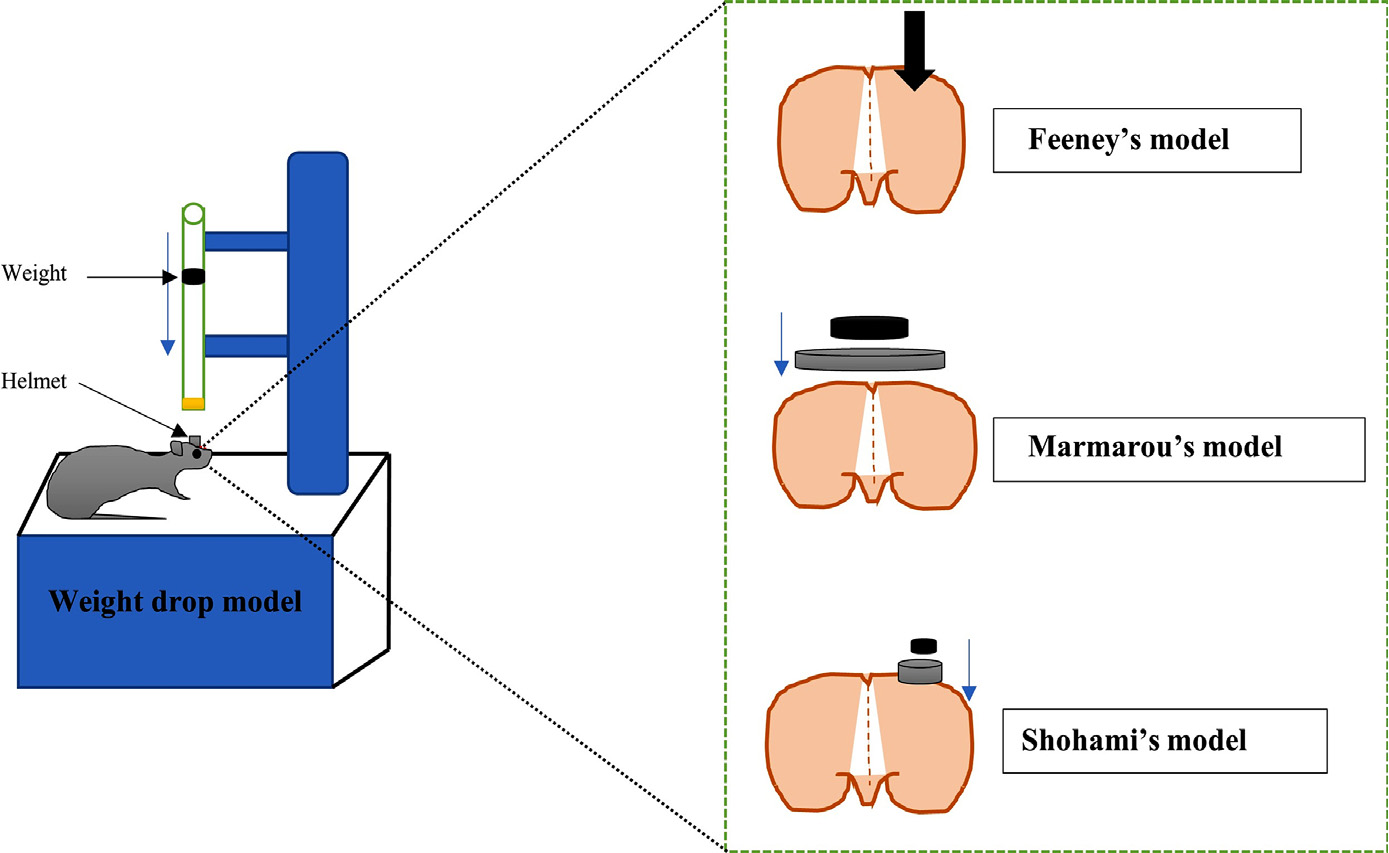
Weight drop model. In Feeney’s weight drop model, weight is to be dropped directly over the footplate resting on exposed dura through craniotomy. This device is used to produce focal cortical contusions. In Marmarou’s weight drop model, injury was delivered by dropping the weight directly upon the helmet. This device is used to produce diffuse brain injury. Helmet is placed to prevent rebound injury. In Shohami’s weight drop model, injury was delivered to one side of the unprotected skull to mimic focal injury.

Diffuse brain injury is associated with high rates of morbidity and mortality. This model is one of the widely used rodent models because of its low cost and high reproducibility. This impact-acceleration model is based on the free-falling of weight over the head by placing the animal on foam surrounded Plexiglas frame with the exposed skull. One stainless steel helmet (10 mm in diameter and 3 mm thick) is placed to prevent possible cranial fracture as well as focal injuries. The trauma device consists of brass weights falling freely over the helmet disc by gravitational forces. The brass weight is connected to produce a falling weight ranging from 50-500 grams from a height of 2 m (Fig. **4**). After the first injury, rebound impact is prevented by moving the foam bed immediately after the injury. The model is useful for evaluating the trauma levels (mild injury and severe injury) with no skull fracture [88].

##### 4.1.1.1 Pathophysiology

This model creates traumatic diffuse axonal injuries, followed by brain swelling [89]. Brain edema is associated with blood-brain barrier (BBB) tearing that stimulates both the activation of microglia and neuroinflammatory cytokines inside the cranial compartment [90]. This model is useful for studying BBB integrity, biomechanical analysis, and molecular mechanisms related to head trauma [91]. Marmarou’s weight drop model appropriately mimics the cases of a car crash and also has been established for the study of repeated head trauma [89].

##### 4.1.1.2 Utilization

The impact acceleration model or Marmarou’s weight drop model is used widely to mimic diffuse axonal injuries without focal lesions to be closely associated with human diffuse traumatic brain injury caused by car accidents or falls, or sudden jolt [92].

##### 4.1.1.3 Strength and Weakness

This model succeeded in replicating human TBI. In this model, severity can be controlled and it showed post-injury neuroscoring very effectively. This model is supposed to show a high mortality rate due to skull fracture, which leads to an increase in the failure rate on acceptance. The major limitation of this model is less reproducibility and variable brain injury [93].

#### 4.1.2 Shohami’s Weight Drop Model

Various animal models have been developed to mimic the close head injury of humans. Mimicking the complexity of human TBI is a very challenging task. Shohami’s group introduced an animal model of close head injury (CHI) by delivering or dropping weight to one side of an unprotected skull in rats. The induction of CHI to the rodent model involved a short duration as no pre-injury manipulation was required. This model deals with more concussions and less contusion. Several experimental CHI represent the same acute hemorrhagic lesions during the post-traumatic phase [90].

Weight drop injury is introduced onto the exposed skull. The mortality or severity of this model is based on using rat or mouse strain [90]. In the procedure of CHI, the impact device head is fixed, and a metal rod of 333 g passes through the cylindrical platform to produce an impact on the head. The weight of the falling rod can be increased up to 1600 g by adding various metal cylinders. At the end of the impacting rod, a silicon tip is fixed with a diameter of 3 mm for mice (32-40 g) and 5-7 mm for rats (200-400 g) [94]. For mild CHI, the weight falling height should be 2 cm, and for severe CHI, the weight falling height should be 3 cm. Falling height greater than 3 cm may result in unwanted skull rupture that can cause respiratory depression in animals. Falling height less than 2 cm may result in very low concussive-like symptoms [90, 95] (Fig. **4**).

##### 4.1.2.1 Pathophysiology

After the impact on the head, several cellular and biochemical changes occur due to the activation and release of neuroinflammatory cascades within the intrathecal compartment. This model also correlates with neurological impairment, blood-brain barrier rupture, and cerebral edema [90]. Currently, neurobehavioral complications, microglia, astrocyte activations, and neurodegeneration have been demonstrated by this CHI model, which also resembles human CHI [96]. One study of CHI demonstrated that histopathological analysis of animals showed significant cell death in different regions (CA1, CA2, and CA3) of the left hippocampal and only CA3 region damage to the right hemisphere [94].

##### 4.1.2.2 Utilization

Shohami’s weight drop model mimics focal blunt injury of human TBI [90]. This model helps to evaluate changes in brain water levels after head trauma. It can closely mimic the clinical consequences of skull injury in combination with direct cortical contusion and focal head injury. This weight drop model can resemble hypoxic, ischemic conditions secondary to initial trauma with human TBI [94]. Khalin *et al.* introduced a modified Shohami model to check the cognitive impairment post-TBI [97].

##### 4.1.2.3 Strength and Weakness

It is easy to create this model and also describe neurological impairment or severity of head injury based on NSS 1 hour post-injury. By this model, cognitive ability can also be reproduced accurately [94].

The major limitation of this model is that it fails to create significant skull injury or unwanted secondary rebound injury [90].

#### 4.1.3 Feeney’s Weight Drop Model

To develop a cortical contusion that resembles a human, this weight drop model is too beneficial. In this model, the weight is directly placed on the intact dura through craniotomy, which causes injuries in the white matter after the first hour of weight drop [98]. The injury, after 24 hours, alters BBB permeability, which is followed by infiltration of immune cells, astrocytes, and microglial cell activation [99]. These inflammatory cells create a necrotic cavity and develop into trauma with a severe contusion in the rat.

This device is mainly mounted on stereotaxic apparatus with a base made up of stainless-steel circular footplate. The footplate is placed just upon the surface of the dura during weight drop to prevent contused cortex from being herniated into the opening. After the craniotomy, a 40 cm length guided stainless steel pipe is placed, and the pipe is perforated at 1 cm intervals to prevent air compression in the tube [98] (Fig. **4**).

In this model, delayed microcirculatory disturbances and cortical spreading depression have been established. Post-traumatic neuronal cell death depends on the severity of impact on the exposed brain [100]. In this model, biomechanics of injury mechanism is similar to clinical human TBI, and this model cannot mimic skull fracture. This model has somehow failed to gain acceptance due to the need for craniotomy and the high mortality rate [98].

#### 4.1.4 Maryland’s Weight Drop Model

This weight drop model is the modification of the impact-acceleration model of Marmarou [101]. This model mimics the frontal impact close head diffuse injuries of human TBI. Diffuse axonal injury is associated with subarachnoid hemorrhage, scattered intraparenchymal petechial hemorrhages, and axonal injuries. In this model, the impact force is applied to the anterior part of the cranium and causes anterior-posterior plus the sagittal rotational acceleration of the brain inside the cranium. In this device, the energy is applied *via* a steel ball (~500 g), which is accelerated due to the gravitational force created by rolling down from an inclined height of 2.1 m. The path of the moving ball is directed by a pair of rails, and the long end of that rail makes a 66-degree angle from the horizontal, which resembles a hockey stick. After completing the path, the ball enters the collecting chambers, where it strikes the coupling arm. The coupling arm passes through a hole in the back wall of the collecting chamber, with the rat lying horizontally just opposite to the hole outside the chamber. When the ball hits the coupling arm, it will allow direct frontal impact without damaging the rat’s facial structure [102].

The model has been reported to cause upregulation of beta amyloid precursor protein, caspase-3 activation, and also neurobehavioral changes due to forces of linear, rotational, and angular acceleration through the device.

Maryland’s model is likely to reproduce several features that may not be carried out by other close head injury techniques. This model is likely to cause an impact on the cerebellum, which is not reported under the Marmarou model. Cerebellum injury through this model also possesses contrecoup injuries, but this mechanism of injury is not also reported under the Marmarou model [103].

### 4.2 Fluid Percussion Model (FPI)

The fluid percussion model of TBI was initially designed to provide a similar effect with focal as well as diffuse brain injuries [104]. This model causes injury to animals by rapidly injecting fluid into the epidural space, and this model resembles clinical TBI in terms of pathological events. A widely accepted fluid percussion model can also reproduce biomechanical, physiological, and neurological responses observed in human close head injury (CHI). This technique produces transient pressure (~20 msec), which is similar to that recorded in the human skull after sudden head trauma [105]. Fluid percussion injury in animals includes contusion, concussion, tissue tearing, subdural hematoma, and hemorrhage, as observed in sports injury [106]. Based on the location of craniotomy, neurodegeneration, neurological impairment, and severity of the injury are assessed.

FPI can be divided according to the position of the craniotomy (sagittal suture). These are divided into midline (centered on the sagittal suture), parasagittal (<3.5 mm lateral to the midline), and lateral (>3.5 mm lateral to the midline) [107]. The midline fluid percussion model was primarily developed for rabbits and cats [108]. Later, it has been introduced in rats also [109]. In rodents, this technique was modified as a lateral fluid percussion model (LFPI). All types of injury can cause significant hypotension that further causes hypertension as well. The strength of injury is based on pressure exposure range and the righting reflex.

#### 4.2.1 Craniotomy

Rats are anesthetized with sodium pentobarbital (50 mg/ kg; i.p.). At first, a bilateral femoral cutdown is performed in femoral venous (for drug administration) and arterial (for blood pressure monitoring) catheters are inserted. On a stereotaxic apparatus, after the exposure of the skull, a 2 mm hollow female Leur-Loc fitting (for the induction of trauma) is fixed with dental cement to the skull. The dura is left intact at the opening. The craniotomy performed at the middle of the sagittal suture is called as midline fluid percussion model (mFPI). Recording electrodes with stainless steel screw are inserted into the skull over the sensory-motor cortex (right) and the nasal bone for recording brainstem auditory evoked potentials (BAERs) as well as electroencephalographic tracings. After performing the procedure, a continuous intravenous infusion of sodium pentobarbital (15 mg/kg/hr) is started for the rest of the physiological studies [109].

#### 4.2.2 Injury Process

**Fig. (5):**
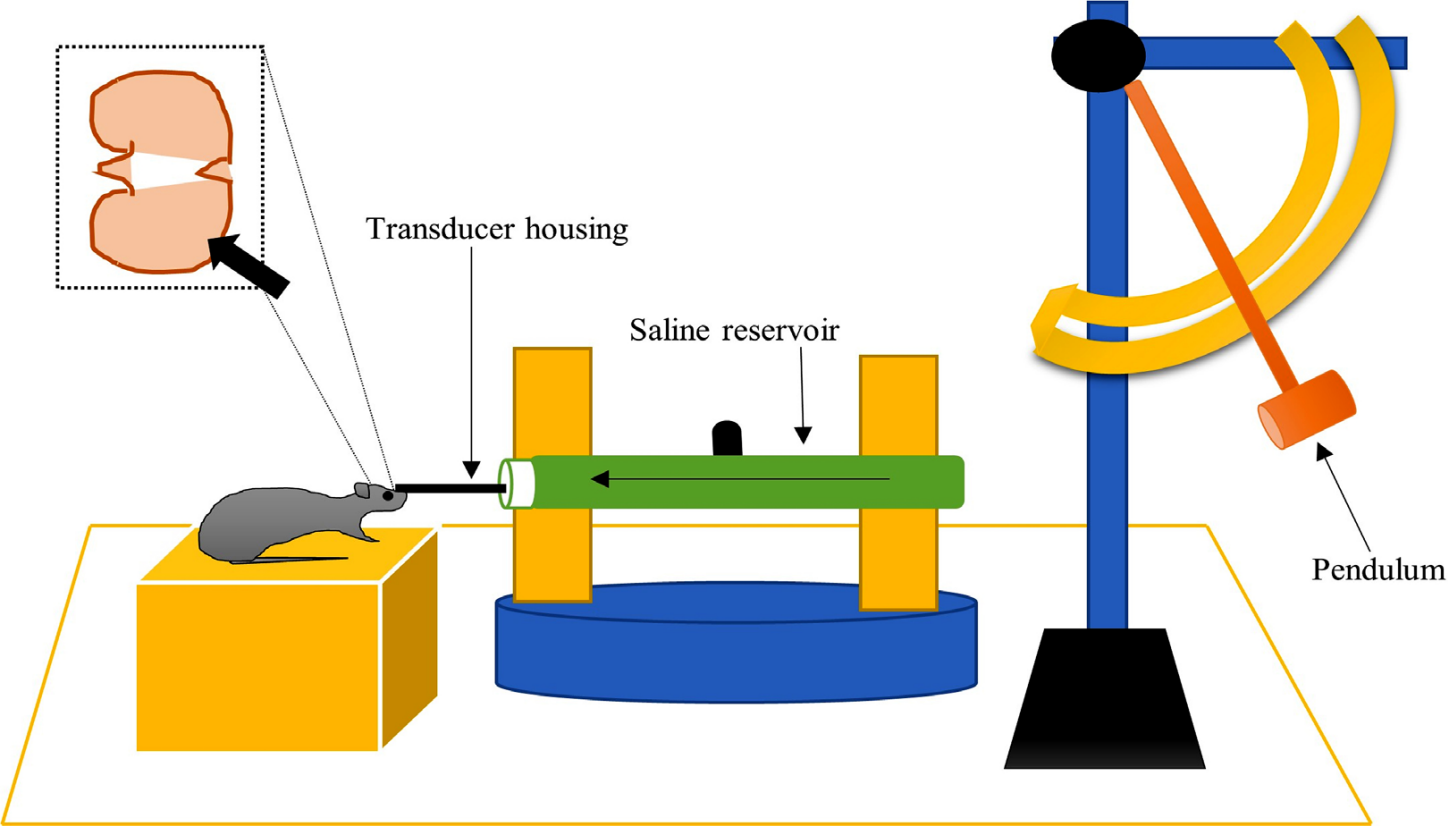
Fluid percussion model. In the fluid percussion injury device, the injury results from the pressure waves in the form of fluid bolus directly on the exposed dura.

The device consists of a Plexiglas cylindrical reservoir, 60 cm long and 4.5 cm in diameter, which is bounded at one side of Plexiglas, cork-covered piston mounted on 0-rings. The other side of the reservoir is attached with 2 cm long metal housing on which one transducer is fitted and connected to a 5 mm tube with 2 mm of inner diameter that terminates with male Leur-Loc fitting. Before the injury, the tube is connected to a female Leur-Loc fitting attached to the exposed dura of the animal. Once the entire tube is filled with isotonic saline (37°C), injury is induced by the pendulum (metal), which hits the device from a predetermined height. Into the close cranial cavity, injection of fluid volume (saline) produces an increase in intracranial pressure of constant duration (21-23 msec). Rapid epidural injection of fluid results in brief displacement and deformation of neural injury. The severity of the injury is regulated by maintaining the height of the pendulum, which causes a variation in pressure expressed as atmosphere (atm). The induced pressure is measured extracranially by a transducer during the time of injury and is recorded on a storage oscilloscope captured on a polaroid camera [109] (Fig. **5**).

Later on, a microprocessor-controlled, pneumatically-driven instrument has also been proposed. This device works through dry compressed air or pneumatic pressure waves, which are used in the form of fluid bolus directly on exposed dura. This model offers control of impact pressure and dwell time of the fluid pulse into the cranium. With this device, the pressure of 2.2-2.4 atm, 2.6-2.8 atm, and > 3.0 atm denotes mild, moderate, and severe injury, respectively. Studies report that this device at a pressure greater than 3.2 atm exhibits a > 65% of mortality rate (Table **1**). This device helps to maintain the animal position during the experimental procedure and can produce acute and chronic TBI features similar to the lateral fluid percussion model of rats.

#### 4.2.3 Pathophysiology

Lateral fluid percussion generally reproduces various types of histopathologically associated human TBI. Based on injury severity, LFP is mainly a focal contusion in the cortex accompanied by intraparenchymal hemorrhage. Skull fracture and surface contusions beyond multiple gyri are the clinical features of moderate to severe human TBI [110]. LFP does not reproduce these features, but intracranial hemorrhage, brain swelling, and damage to gray matter are the major pathophysiological hallmarks of both human TBI and LFP [111]. The release of glutamate and aspartate and activation of glutamate receptors result in Na+ influx, K+ efflux, and Ca^2+^ influx into the cell [112, 113], resulting in cell swelling and excitotoxic cells destruction after LFP injury. In rodents, the damage progresses to apoptotic and necrotic neuronal death after LFP. Moreover, the levels and protein distribution involved in cell death, inflammation, and synaptic transmission are also impaired by this model [114].

#### 4.2.4 Utilization

The fluid percussion model is used widely to mimic the several clinical features of human TBI (deformation of the brain, brain concussion, contusion, including sports-related TBI) [106]. The lateral fluid percussion model subsequently reproduced human brain injury in terms of physiological function, behavioral features, and neuropathological outcomes.

#### 4.2.5 Strength and Weakness

The success rate of FPI is higher than other available TBI animal models. The FPI model is well characterized, and the severity of brain hemorrhage can be easily adjusted. In this model, mechanical factors, including velocity and depth of impact, can be easily regulated [115]. So, it is most useful for the evaluation of biochemical outcomes of TBI. Recently, fluid percussion waveforms can easily be controlled by the computer-controlled LFPI device [116].

Although it has a high success rate, midline FPI showed a higher mortality rate due to brainstem compromised apnea (pressure greater than 2.5 atm showed 34% of mortality) [117]. FPI model requires craniotomy before the injury. This device needs calibration of pressure since an air bubble may originate inside the fluid, which can result in a variable degree of the lesion [109].

### 4.3 Controlled Cortical Impact Injury

Controlled focal mechanical cortical deformations injury or controlled cortical impact (CCI) is a mechanical injury model of TBI developed approximately 30 years ago to determine biomechanical brain tissue properties. The experimental pneumatic impactor model was first developed by Lighthall and his colleagues [118], and then this procedure was introduced to rats by Dixon and colleagues for the betterment of biomechanical control [119]. The model was firstly used for ferrets and then employed in rats, mice, swine, and monkeys. Several new applications of this model have been approved for studying different human TBI consequences, including close head injury and brain deformation [119]. In the last 30 years of TBI research in CCI models, some progress has been made. Initially, this model successfully explored the biomechanical and physiological changes, but later on, the model was expanded for the cellular and histopathological characterizations to find secondary injury due to CCI. Traditionally, before the induction of cortical impact on the animal brain, craniotomy is done. This device mechanically transfers energy onto the exposed dura matter and damages the cortex as well as subcortical structures in case of severe injury [120].

Mainly, there are two main types of CCI devices available, pneumatic and electromagnetic. From the past till now, the CCI model still uses pneumatic devices, but as an alternative, electromagnetic devices are gaining popularity due to their lower cost [120].

#### 4.3.1 Pneumatic

**Fig. (6):**
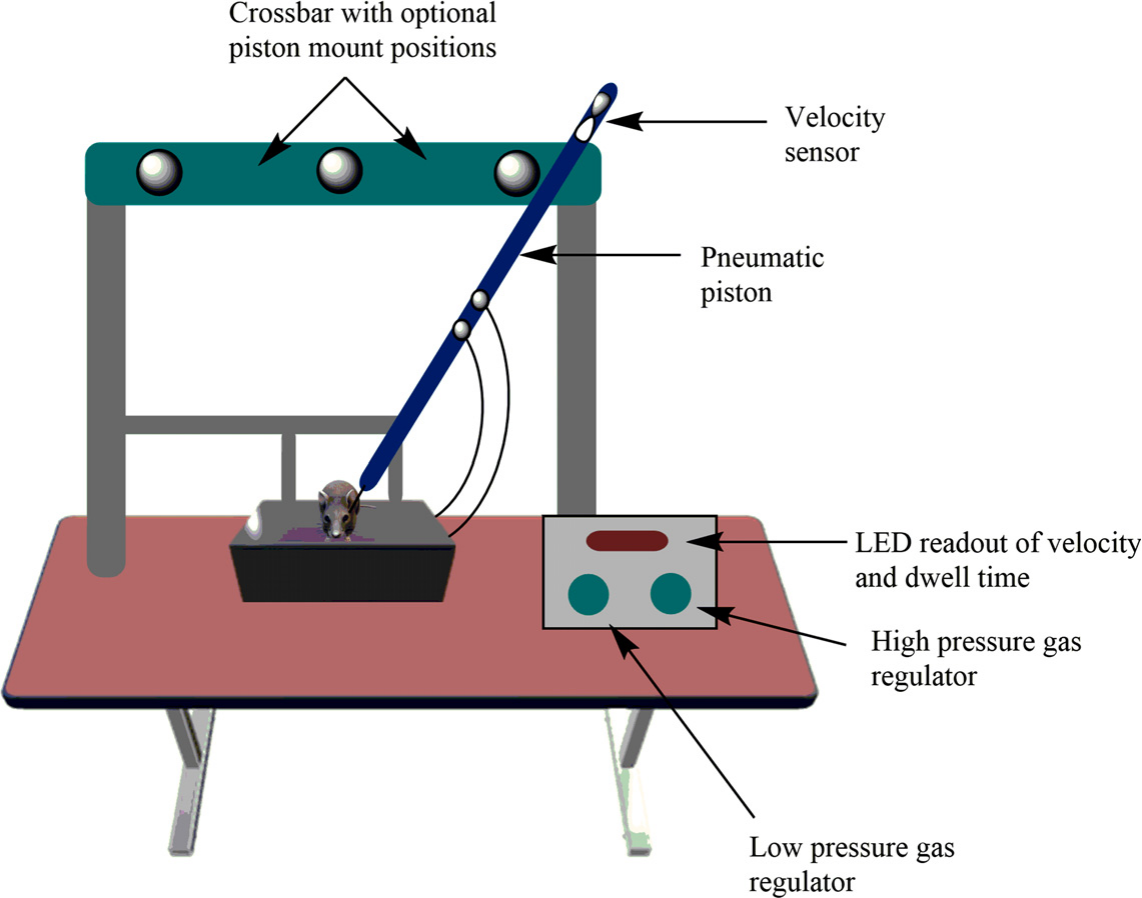
Controlled cortical impact injury model. Model uses pneumatic or electromagnetic driven piston to induce cerebrovascular injury at a known distance and velocity.

Since the first development of CCI by Lighthall and his colleagues, the device has been powered by pressurized gas (*i.e*., pneumatically driven). This device is used widely to study the pathophysiology of traumatic brain injury as well as to test novel therapies [121]. The device includes a cylinder, which is mounted to a crossbar and consists of different mounting positions on the crossbar, so the impactor can be angled in a vertical position respective to brain tissue. The device has a small-bore reciprocating double-acting pneumatic piston with a maximum adjustable stroke length of 50 mm. The lower rod has an impact tip, which is either round or flat edge with a diameter of 5-6 mm, and the sensor system is connected with the upper rod for measuring velocity. On the exposed neuronal tissues, the piston propels a tip, and in the case of close head injury, the tip hits the intact skull. The pneumatically driven impactor tip is based on the predetermined velocity, depth, and tissue deformation. The depth of the tissue injury as well as the velocity of the rod vary from lab to lab. For example, Yu *et al.* reported a study in which they induced injury to the brain of a rat at a depth of 0.5 mm for mild TBI, 1.0 mm for moderate TBI, or 2.0 mm for severe TBI (velocity 6.0 m/s) [122], while Washington *et al.* introduced brain injury in mice at a depth of 1.5 mm for mild TBI, 2.0 mm for moderate TBI, and 2.5 mm for severe TBI (velocity 5.25 m/s) [123]. After the brain injury, they reported 10%, 25%, and 40% tissue injury in rats and 5%, 15%, and 30% tissue injury in mice, respectively. Wang *et al.* introduced mild, moderate, and severe injury in mice at a depth of 0.2 mm, 1.0 mm, and 1.2 mm (velocity 3.5 m/s), respectively [124]. After the critical analysis, the proposed principle for brain injury is recommended as follows: for mild injury, the depth of injury should be less than 1.0 mm and velocity should be less than 4.0 m/s; for moderate injury, the depth should be in between1.0-1.05 mm and velocity in between 4.0-5.0 m/s; and for severe injury, the depth should be greater than 2.0 mm and velocity in between 5.0-6.0 m/s (Fig. **6**) [125].

#### 4.3.2 Electromagnetic

This device has recently gained more popularity and shares most of the features of already developed pneumatic devices. The electromagnetic device is traditionally used in combination with a stereotaxic frame, which facilitates impactor angle adjustment. This device is smaller in size and is easy to carry or move. Commercially, there is a varying number of tip sizes and shapes available (flat, round, beveled). For different animals, different tip sizes are available. For example, 3 mm tips for mice, 5-6 mm tips for rats, 10 mm tips for ferrets, and 15 mm for pigs. Like the pneumatic device, the electromagnetic device also reproduces brain trauma and brain deformation [120].

#### 4.3.3 Pathophysiology

CCI model mimics the histopathological changes seen in TBI patients. Gross histopathological changes in the CCI model include blood-brain barrier (BBB) disruption, cortical contusion, hippocampal cell, and brain volume loss. Human TBI patients experience secondary injury with cellular markers, like apoptosis, inflammation, and oxidative stress [126]. Several TBI consequences have been found to persist for more than 2 weeks and move to chronic neuronal insult. In the chronic stage, CCI is known to show chronic ventricular enlargement and shrinkage of white and grey matter [126], and also cause necrosis and intraparenchymal injury [127]. The pathological changes after CCI include lesion volume expansion, microglial activation, hippocampal neurodegeneration, myelin loss, axonal injury, and ventricular enlargement.

#### 4.3.4 Utilization

Recently, the CCI model has been adapted to mimic human close head injury, including contusions. Individuals who are in the military or performing sports activities experience a close head injury, which has become the area of research interest [128].

The low rate of mortality after CCI makes it useful to find long-term pathological changes in TBI. In the CCI model, behavioral deficits include memory and learning impairment, and motor changes. CCI injury can cause emotional changes that can be measured in forced swim tests and elevated-plus maze [123].

#### 4.3.5 Strength and Weakness

The controlled cortical impact model has a higher degree of success rate as this model has control over injury parameters, including depth of impact, velocity, duration, and site of impact. This model is highly acceptable as it shows high reproducibility with a low mortality rate. Also, this model lacks a risk of a rebound injury [129, 130].

Although this model has aforementioned strengths, it also holds some limitations, like mechanical variation and diffuse injuries. The working of this model is complex and also not accurate to demonstrate clinical consequences.

### 4.4 Blast Injury Model

Many militaries and sports personnel who undergo severe internal injury without external blood loss are diagnosed with TBI. Mild blast-induced TBI is caused by exposure to explosive blast (low level), which is a significant concern behind combat force health systems. Currently, several animal models have been established to mimic different clinical consequences of blast injury. To elucidate primary blast wave injury in the brain, rodents, swine, and primates are preferred. Blast injury causes diffuse axonal injury either by compressed air or by the detonation of explosives. Using these blast waves, physiological, neuropathological, and neurobehavioral outcomes are assessed [131].

#### 4.4.1 Shock Tube Injury with Compressed Air

The laboratory-designed shock tube model uses primary blast wave energy inside a cylindrical tube, which is seen in free field explosions. The McMillan blast injury model consists of a cylindrical metal tube with an internal diameter of 12-inch separated by a 19-inch expansion chamber and a 2.5-feet compression chamber. The metal shock tube consists of a gas compressor chamber, and at the end of this tube, at the metal diaphragm, a blast wave energy propagation tube is attached [132].

In this model, after giving the anesthetics to animals, they are restrained in a holder outside the propagation tube. To protect the thorax and abdomen, animals are attached to Kevlar thoracic protective vest. Compressed air is given or compressed helium is used for filling of gas, and compression is done until the diaphragm ruptures. The blast wave is created by releasing compressed air through the cylindrical metal tube. The duration of the pulse is usually longer, but the peak pressure is relatively low [132]. This shock tube injury has a lack of quaternary blast effects, which can be considered an advantage or limitation of this model [133].

#### 4.4.2 Blast Tubes for Explosives

**Fig. (7):**
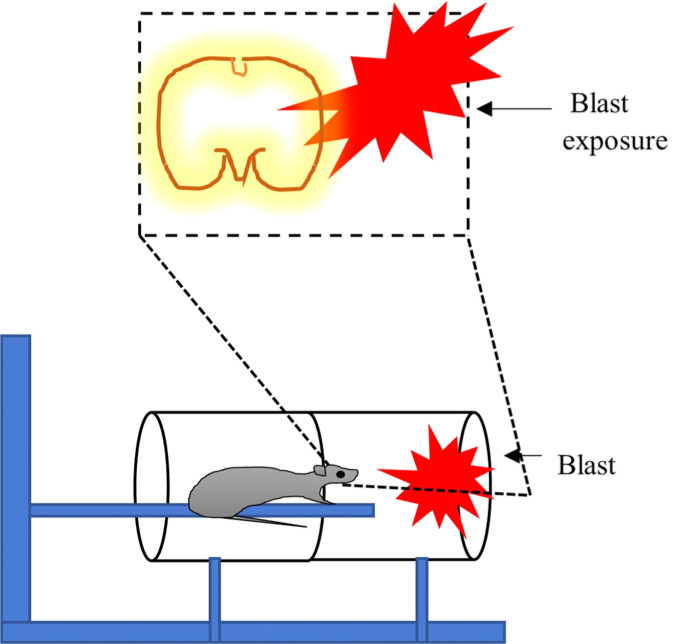
Blast injury model. In this model, non-electrical ignition is done by pentaerythritol tetranitrate (PETN) explosive, resulting shock waves at the surface of the animal at a duration of 1-2 ms.

For primary blast injury experiments, a 1.5 m long metal tube is used, which has been demonstrated by Clemedson [134] for experimentation on rabbits. Later, this model has been modified for rats [135] by keeping them in the holder and then employing a non-electrical igniter for detonation. The animal is placed in a net holder with a 1 m distance from charge (1-2 g of pentaerythritol tetranitrate, PTEN explosive), which results in a shock wave at the animal surface with a duration of 1-2 ms and peak pressure of 136-236 kPa. Pressure greater than 236 kpa showed bleeding from airways (>50% of animals) (Fig. **7**) [136].

Liu *et al.* designed a model in which, before the explosion, anesthetized rats were placed in a soft bed under miniature spherical explosives. Miniature explosives can be adjusted 2 mm over the scalp and 3 mm right of the center point between bregma and lambda. For moderate and severe injury, 2.5-mm and 3.0-mm diameters of explosives are adjusted. Explosives contain PTEN, and throughout the study, the parameters are constant with charge density of 1.50 g/cm^3^, detonation velocity of 75000 m/s, detonation pressure of 22 Gpa, and the TNT of 15.6 mg and 27.0 mg in 2.5 mm and 3.0 mm of spherical exploders, respectively. This model can create blast injury in 8 animals at one time by using an electric detonator [137].

#### 4.4.3 Pathophysiology

Rat model with blast injury mimics human blast exposure that causes mild traumatic brain injury and subsequently activates secondary pathogenic pathways [132]. Non-impact blast injury is characterized by diffuse cerebral brain edema, extreme hyperaemia, and delayed vasospasm [138]. Blast exposure to rats showed diffuse axonal injury within the first 14 days. After the exposure to blast waves to the head, the rat’s brain showed significant neurological problems; also mild blast waves to the brain can subsequently increase intracranial pressure and can cause cognitive deficits [139].

Cernak *et al.* highlighted the pathophysiological consequences and behavioral dysfunction through their multi-chamber shock-wave model [140]. They investigated blast wave consequences in mice (both supine and prone positions) that generated mild, moderate, and severe injury at 103 KPa, 124 KPa, and 190 KPa, respectively (Table **2**).

#### 4.4.4 Utilization

This model has been used to check the severity of axonal brain injury after blast TBI. This model helps to evaluate various behavioral changes. The lesion after an overpressure blast represents shear injury that is noticed within military combat forces. Based on the position of animals (prone or supine) in shock tubes, injury severity can be evaluated that is seen on the military battlefield [141]. This model also mimics transient vestibulomotor deficits and persistent orofacial pain that is seen in combat forces after blast [142].

#### 4.4.5 Strength and Weakness

The biomechanical injury mechanism is similar to that seen in combat force TBI. This model is not well standardized, and it needs further standardization. Blast TBI models of rats may or may not show skull fracture [133].

### 4.5 Penetrating Ballistic Brain Injury Model (PBBI)

Penetrating brain injury is caused by motor vehicle accidents and mostly by military combat forces or people in the most violent areas [143]. Pathological consequences are tissue damage, inflammation and bleeding, subsequently causing brain swelling. Currently, few animal models have been approached for this type of brain injury, using cats, dogs, monkeys, sheep, and rats. Larger animals are used as they mimic human physiology and are closer in size to humans, whereas small animals would be beneficial to analyse biochemical and histopathological changes. In this model, an inflated balloon is placed into rat brain parenchyma and, after a high-speed missile pierces the balloon, tissue damage is produced by creating a cavity. This model shows a lack of repeatability and a high rate of mortality in rodents. Risling *et al.* and Plantman *et al.* developed a new model in which a probe is introduced rapidly into brain parenchyma and means to be stopped at a depth of 5.5 mm and causes laceration in brain tissue with changes in intracranial pressure [136, 144]. Brain tissue damage causes white matter degeneration, hemorrhage, and gliosis.

**Fig. (8):**
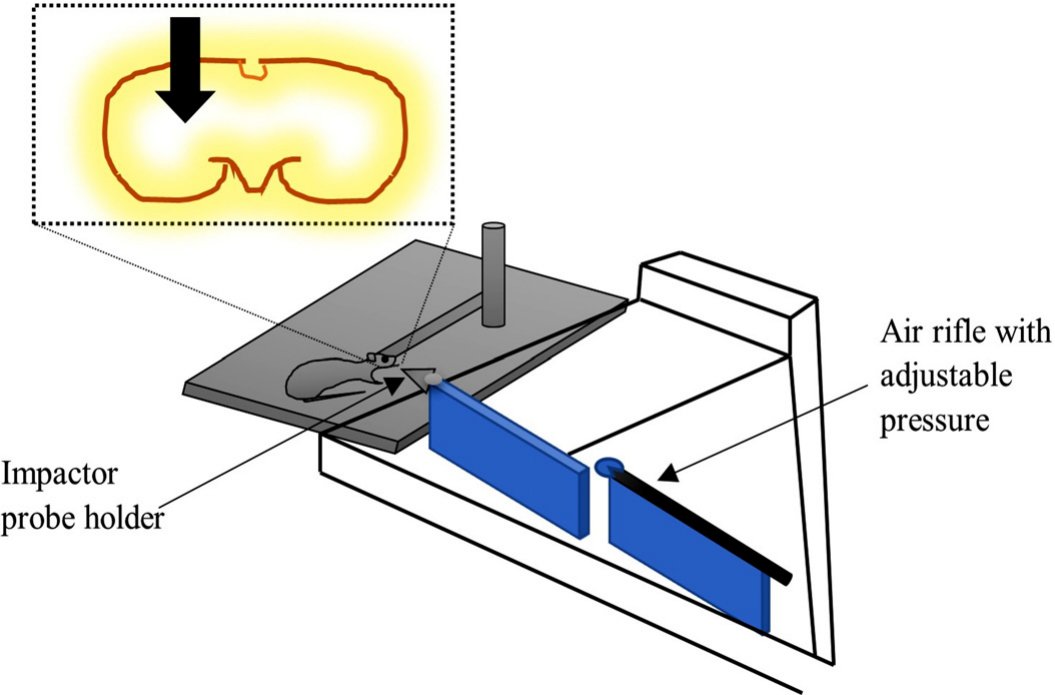
Penetrating ballistic brain injury model. The penetrating brain injury involves transmission of a metal rod with high energy to the skull. This model ruptures blood brain barrier and brain edema formation.

In this model, first of all, the anesthetized rat is placed in a stereotaxic frame, and a drill is done to create a burr hole 2.75 mm in diameter. In principle, brain injury is achieved after a lead bullet is accelerated through an air-driven accelerator and made to hit the probe (second target) that penetrates the brain parenchyma. The air-driven accelerator (air-driven rifle) consists of a 5.5 mm barrel that maintains air pressure from an air tank. The rifle is mainly mounted to a base for adjusting air pressure at 50 bar connected to a pressure gauge once the bullet strikes, and then some of the kinetic energy is transferred to the probe, which helps in the penetration of the brain with high velocity. At 50 bar air pressure, the maximum velocity during penetration is 110 m/s (Fig. **8**). After induction of injury, the animal is returned to the heating pad and reconnected with a pulse-oximeter. After the brain injury, various parts of the brain, including lateral and midline parietal cortices, hippocampus, and corpus callosum, can be observed to be damaged.

Cernak *et al.* showed a novel mouse model of penetrating brain injury where they considered loading pressure of 35-bar for mild injury, 50-bar for moderate injury, and 100-bar for severe injury [145]. After induction of injury, they analyzed glial activation in the cortex of mice and showed motor function alterations.

#### 4.5.1 Pathophysiology

This penetrating model shows various pathophysiological characteristics as likely reported in other TBI models, including brain edema, increased intracranial pressure, neuroinflammation, and white matter degeneration [146]. In contrast to other models, the PBBI model causes intracerebral hemorrhage due to a penetrating type of injury and also forms a cavity temporarily. This model causes focal injuries and also highly replicates contusion to human conditions. The model serves as a relevant model that can mimic moderate to severe brain injuries.

#### 4.5.2 Utilization

This model has been found to be successful in studying blood-brain barrier (BBB) permeability, brain swelling, cognition and motor dysfunction [147].

#### 4.5.3 Strength and Weakness

Biomechanics of injury mechanisms is similar to that seen in human traumatic brain injury. This model does not differentiate all clinical phases of tissue injuries and has a high failure rate. This model allows for creating injuries at a straight angle to the direction of insult [147].

### 4.6 Zebrafish Traumatic Brain Injury Model

Zebrafish (*Danio rerio*) is a good vertebrate model of different neurological diseases for understanding cellular and gene function because of its high genetic homologies to the human genome [148]. The comparison between zebrafish and human protein-coding genes demonstrated that 71.4% of human genes have at least one zebrafish ortholog, and among those genes, only 47% of human genes have a one-to-one relationship with zebrafish orthologs [149]. For these types of characterizations, nowadays, zebrafish has achieved much success as a research model in various neurological as well as genetic disorders.

In the subventricular zone (SVZ) of the adult mammalian forebrain, neuronal stem cells act as an active source of neurons for neuronal tissue repair after brain trauma or head injuries, including traumatic brain injury and ischemic stroke [150]. For non-mammalian animals (birds, fish, and reptiles), the telencephalic ventricular zone generates neural precursor cells that share the same characteristics with the neural progenitor cells in the SVZ of mammals [151].

**Fig. (9):**
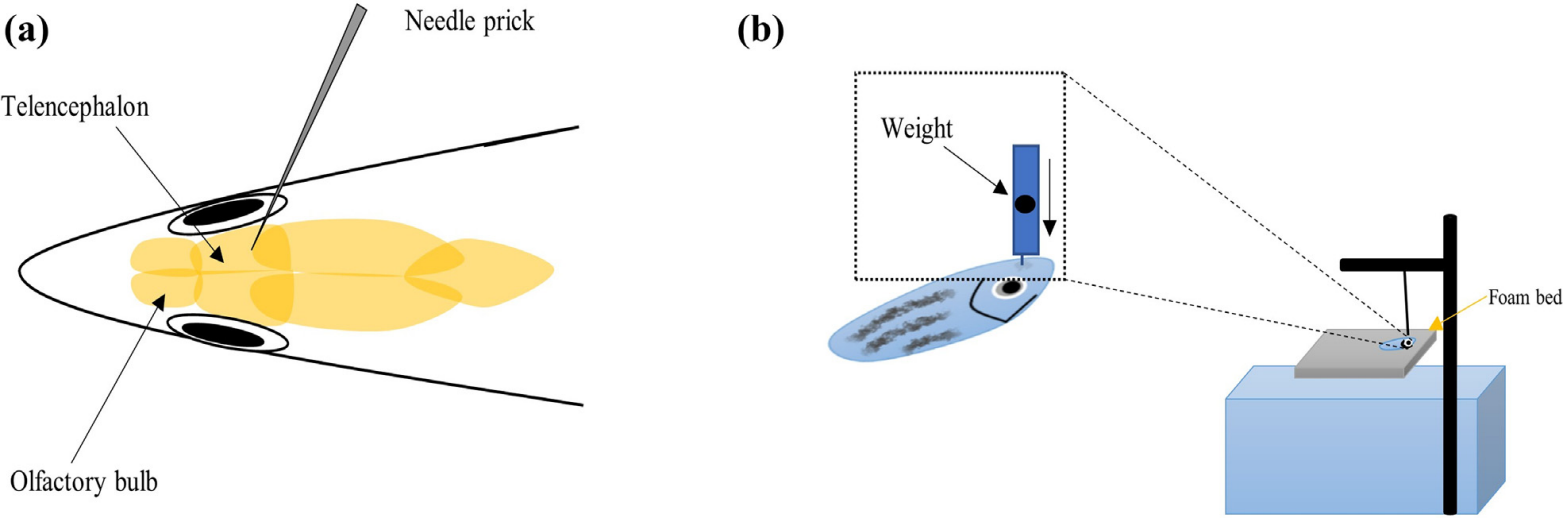
(**a**) Zebrafish TBI model. In this model, telencephalic injury is introduced by inserting a needle into the dorsolateral domain of the telencephalic hemisphere. (**b**) Zebrafish TBI model. The mild traumatic brain injury weight drop model in the brain of adult zebrafish. This model uses non-penetrating injury and mimics diffuse brain injury.

Several TBI models in adult zebrafish have been approached to find behavioral alteration with brain injury. Hannah *et al.* demonstrated an open-head acute TBI in adult zebrafish. In this, firstly, zebrafish are anesthetized and secured in an agarose mold. In the left brainstem and midbrain, a deep puncture is performed to make an injury by inserting a blunted sterilized syringe needle (26 3/8 gauze, 0.2 mm) through the foramen magnum at the skull base (Fig. **9a**). After the fish are allowed to swim in water and resuscitation is done *via* mechanical water dispersal, altered swimming behavior changes are observed. After the injury, approx. 70% of recovering animals swam in a counterclockwise direction, whereas 30% of injured zebrafish swam in the corkscrew direction. The survived TBI animals showed improvement in swimming behavior after 3-7 days of acute injury [152].

Kishimoto *et al.* showed neuronal regenerations in adult zebrafish (transgenic) model of brain (telencephalic) injury [153]. For telencephalic injury, first of all, adult zebrafishes are anesthetized with tricaine, followed by insertion of a sterilized syringe needle (27 gauge) into the dorsolateral domain of the telencephalic hemisphere to create a wound in the telencephalon (< 0.1 mm deep stab). After creating the injury, animals are allowed to recover in water. The researchers found that the model may be a useful tool for *in vivo* drug screening that promotes neurogenesis and may be used for the treatment of traumatic brain injury (Fig. **9b**).

Mychasiuk *et al.* established a novel TBI model of zebrafish in which they have shown genetic pathways of neuroregeneration after brain injury [154]. In this study, they only mimicked the rodent weight drop model in adult zebrafish. Only moderate to severe TBI is approached in adult zebrafish.

This apparatus consists of a stand in which a 10 cm plastic tube (outer diameter 0.5 inches and inner diameter 0.187 inches) is attached. A water tank (2.8 liters) is filled with system water, and then a foam block is adjusted above the recovery water tank to act as a cradle for fish. The tube is adjusted just one cm above the cranium of the fish. After being anesthetized with 0.02% tricaine -S, fish are quickly placed on a foam bed at a position with the dorsal side erect and ventral side to the foam. The upper side of the head is positioned just below the tube to ensure that the ball would strike the cranium. Afterward, a single 4.5 -mm ball bearing, with a mass of 0.33 gram and weight of 0.0032 N, is dropped. With a maximum speed of 1.5 m/s in 0.073 seconds, the ball bearing strikes the cranium with a maximum impact energy of 35 mJ. Then, the fish are quickly placed in the recovery tank, and after recovery, they are analyzed for differentially expressed genes at 3 and 21 days post-injury. The spatial memory of fish is also checked for shoaling. The result highlighted that mild TBI fish took a long time to find out the correct spatial location of shoaling than control groups on the TBI induction day as well as 1 and 3 days post-injury [155].

#### 4.6.1 Pathophysiology

Telencephalic injury after mechanical damage (from 4 h post-lesion to 6 h post-lesion) causes cell death in the brain parenchyma and periventricular zone detected by TUNEL staining [156, 157]. TUNEL-positive cells showed several features of necrotic and apoptotic cells post-injury. Kroehne *et al.* observed the cell death in the parenchymal as well as the periventricular region to still be present on day 1 of post-lesion but it returned to normal level at day 3 [156]. Whereas, Kyritsis *et al.* observed decreased numbers of apoptotic and necrotic cells at 3 days of post-lesion [156]. Additionally, mechanical injury causes strong brain edema after one day of post-lesion, but the cerebral volume is reduced at 7 days [156].

#### 4.6.2 Strength and Weakness

This model is effective yet inexpensive. The mild TBI model of zebrafish will surely be beneficial for the neuroscience community to research zebrafish in response to injury [155].

There is a lack of understanding of how the zebrafish brain shows behavioral changes, and despite having high similarities with humans, there are still some differences that create some impact on studies [158].

### 4.7 Drosophila Model of TBI

*Drosophila melanogaster* is used nowadays as it has several advantages, including a short lifespan, ease of maintenance, low cost, and similarity with human anatomy [159]. Drosophila’s life cycle has 4 morphological stages, such as embryo, larva, pupa, and adult [160]. About 70% of the total fly genome (13,500 genes) is recognized in human diseases that involve Drosophila homolog [161]. Flies have some analogous internal organs to humans, including heart beat, adipose tissue (equivalent to the liver), a tubular network (analogs to the lung), a complex brain (surrounded by the barrier), and a nervous system [162]. These factors implicate that Drosophila is a great model in the brain research area.

The protocerebrum, deutocerebrum, and tritocerebrum regions of Drosophila are homologous to the forebrain, midbrain, and hindbrain of humans, respectively [163]. The brain of the Drosophila is mostly similar to mammals concerning the similarity of neurons and neurotransmitters. Molecular and cellular processes are involved in neurodegenerative diseases, like oxidative stress, which is also seen in Drosophila. Brain injury in flies has been approached by using high-impact trauma (HIT) device, which mainly exhibits immune response activation, ataxia, neuronal degeneration, and death [164]. Moderate and severe brain injury in flies has been generated by using a homogenizer that exhibits inflammatory cascades as well as tau phosphorylation and sleep pattern alteration. As there are various morphological consequences between flies and mammals, flies mostly show behavioral impairment, as also seen in the mammalian TBI model. Method and injury models are depicted in Table **3**.

#### 4.7.1 High-impact Trauma Device

**Fig. (10):**
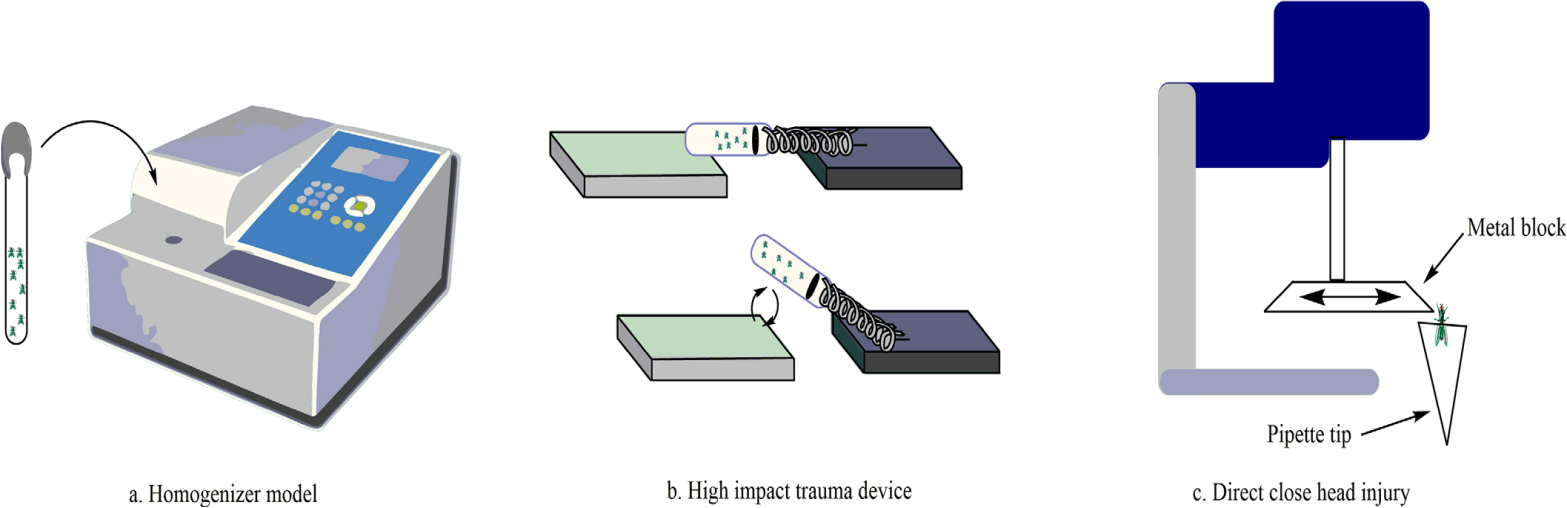
Drosophila TBI model (**a**) Homogenizer model (**b**) High impact trauma device (**c**) Direct close head injury.

To induce trauma in the Drosophila brain, Katzen-berger *et al.* have generated a high-impact trauma (HIT) device [165]. This HIT device consists of a metal spring attached to one end of the plywood board and another end adjusted with a polyurethane pad. Unanaesthetised flies are taken in a plastic vial and enclosed with a cotton ball to the free end of the spring. After the deflection and release of the spring, the vial rapidly hits polyurethane pad, and a mechanical force is accounted to the flies as they strike one side of the vial wall to another wall. Individual flies contact different part of the vial with their different brain region, so the primary injury varies from one fly to another fly in the same vial. This model mimics the close head injury common within human TBI (Fig. **10a**) [166].

The strength of this injury through HIT devices can be adjusted by changing the number of strikes as well as spring deflection. From an angle of 90°, this device produces an impact velocity of ∼3.0 m/s (6.7 miles/h) with an average force of 2.5 N. Loss of motor function followed by ataxia is also seen in human concussive injury, which proves that HIT device can inflict trauma to flies brain [164].

#### 4.7.2 Bead Ruptor

A highly reproducible inflicting TBI model is introduced to a large number of flies, called Omni Bead Ruptor-24 Homogenizer platform. A defined injury paradigm is introduced to the flies by altering the intensity of injury (meters/second, [m/s]), duration of injury (s), and the number of injury bouts. Briefly, 10 flies are kept in an empty tube that is placed individually in the Bead Ruptor. To introduce TBI, flies are subjected to a single injury for 5 seconds with different intensities. It is determined that intensities with 5.0 m/s and greater show nearly 100% mortality within one day, whereas intensities with 4.35 m/s show ~10-25% survival. In low intensities, nearly all flies survive after one day of injury. Intensities with 2.1 m/s are classified as mild traumatic brain injury. Based on injury bouts and intensities, different features like different injury sensitivity, immune system upregulation, and tau phosphorylation are introduced to Drosophila (Fig. **10b**) [167]. Also, TBI in flies tends to show alteration in sleep/cycle-related behaviors similar to mammals.

#### 4.7.3 The Close Head Injury Model

Van Alphen *et al.* designed a closed head injury device to induce head-specific traumatic brain injury in Drosophila [168]. In this model, non-penetrating strikes are directly introduced to the unanaesthetised Drosophila. This model mimics various consequences after inducing TBI, including increased mortality, motor control impairment, sleep/cycle impairment, and neurodegeneration. Existing Drosophila TBI models only introduce impact to the whole body, but this model only causes brain injury.

In this model, W^111B^ male flies are placed in a P200 pipette using an aspirator, and this device introduces trauma to the head by transferring current through a solenoid (delivers 8.34 Newtons of force) that causes forward movement of the brass block to create blow to the fly head. Flies are supposed to be struck 1 time, 5 times, and 10 times (1 strike per second) to observe the effect of the blow. No mortality is observed in 1 strike in 24 hours, but the mortality rate increases with a higher number of strike times (Fig. **10c**).

#### 4.7.4 Pathophysiology

Accumulation of free radicals causes the production of reactive oxygen species. Oxidative stress is linked with the propagation of neurodegenerative disorders following TBI. 24 hours of post-TBI in *Drosophila* showed cyclooxygenase disruption, which leads to oxidative stress and decreased ATP propagation [169]. An increase in antioxidant demand and increased mitochondrial aging, the key symptoms of oxidative stress, have been reported in post-TBI of flies [170, 171]. CNS axonal degeneration and peripheral nervous system are linked with *Drosophila* and human TBI. Within minutes of TBI in *Drosophila,* the retraction bulbs and filopodial sprouts are reported in the region of proximal axonal fragments [172]. In response to axonal injury in CNS, innate immune pathways (JNK and JAK/STAT) present in *Drosophila* are activated [173]. Cell death after TBI in flies is limited to brain vacuole formation, whereas the role of apoptosis and necroptosis is not well clarified [174]. In response to stress, fly mitochondria also swell up as in mammals, demonstrating the involvement of mitochondrial membrane permeabilization in apoptotic signaling pathways [175].

#### 4.7.5 Strength and Weakness

*Drosophila* TBI model showed inflicting mechanical injury to flies similar to outcomes characteristic of brain injury in humans. Outcomes include temporary incapacitation, ataxia, innate immune response activation, and neurodegeneration. Also, these models relate the mortality risk factor of flies to humans [176].

Rather than a high success rate, many limitations have been encountered. Firstly, the important target genes for human TBI may not be present in *Drosophila* [177]. Secondly, there is a difficulty in drug delivery in this model, and one cannot study the effect of the drug in this species and predict the therapeutic effect in humans.

### 4.8 *In Vitro* TBI Models

These models can play a major role in establishing and understanding the mechanism of traumatic brain injury at the tissue level after induction of injury. The goal of *in vitro* study is to find out the pathophysiology of TBIs through experiments and evaluate different parameters sustained by damaged brain areas. From these models, several signaling cascades, such as apoptotic or cell signaling, are studied [178].

In the *in vitro* models, there are chances of studying dissociated cells or whole-brain tissues. These models are effective in controlling experimental variables, so that confused factors can be removed very easily. *In vitro* models are cost-effective and show a high-throughput readout in neuroprotective agents screening. Along with advantages, there are some limitations to *in vitro* model study. First, the behavior of tissue and cells may differ in *ex vivo* in response to injury. Another technical limitation is that the dissected tissues already have undergone injury (dissection process), which may alter tissue response after experimental injury. Another limitation is that the tissue viability is just eight (8) hours [179]. Another limitation that persists mostly is that the *in vivo* extracellular environment is different from the *in vitro* extracellular environment.

#### 4.8.1 Mechanical Damage

This model causes brain damage sustained by cerebral parenchyma after an impact or penetration of an object in the tissue. This model can result in damage directly either on brain tissue or isolated cultured tissue. Mechanical damage can be induced in tissue using a knife, needle, punch machine, or any sharp object, which results in primary injury and subsequently cell loss. After the injury, necrotic as well as apoptotic cell death is seen, and afterward, a series of metabolic cascades (mostly caspase-3 apoptotic pathway) become activated [178]. This model has been tested with simultaneous inhibition of NMDA receptor and caspase-3 activity, showing neuroprotection. Also, the mechanical injury model has been tested with different cyclic dipeptides, and these peptides have shown neuroprotection against injury [180].

##### 4.8.1.1 Success and Failure

This model has been shown to induce consistent injury in neuronal cells, and this model is highly reproducible. Mechanical injury induced by punch showed immediate cell death followed by activation of different cellular and molecular cascades [181].

The limitation of this model is that mechanical parameter evolution has not been established yet, and the severity of the injury cannot be detected properly as the injury is measured by the amount of cell damage only. This type of injury only mimics a small percentage of human clinical outcomes [182].

#### 4.8.2 Compression Induced TBI

This model shows clinical outcomes of traumatic brain injury induced by weight drop in the skull of rodent models. In the isolated cells, a platinum cylinder (300 mg) is dropped from a height, and at the end, a certain pressure is exerted on the cultured tissue that causes damage throughout the cell [183].

The major limitation of this model is that it fails to measure the degree of tissue injury after compression.

#### 4.8.3 Spasticity

Until now, within different *in vitro* models, this model has been used widely for research on traumatic brain injury. This model truly mimics the tissue deformation of cerebral parenchyma by circular hollow indenters under a closed loop. Various *in vitro* models have been approached that use compressed air to induce injury in the cultured tissue cells [184]. Another model has been established, which creates an opposing force from a different angle. This biaxial system model controls the mechanical parameters using computer software [185].

##### 4.8.3.1 Strength and Weakness

This model is highly reproducible, and it can be applied to the hippocampus and cortex very specifically. The major limitations are the high variability and heterogeneity in some cases. In *in vivo* model, swelling of the lateral ventricle is seen near the hippocampal region, but in this *in vitro* model, the aforementioned factor is not seen here [186].

#### 4.8.4 Chemical Injury

In the mammalian central nervous system, glutamate is the major excitatory neurotransmitter, and in gray matter, it is present in a concentration of millimolar. The efficiency of glutamate transmission depends on the proper functionality of a plethora of receptors and transporters situated both on neurons and glial cells. Excitotoxicity is a complicated process activated by glutamate receptor activation, resulting in neuronal cell death [187]. The term excitotoxicity is referred to as the ability of glutamate and its agonists to show neuronal death. Neuronal death caused by the excitotoxic agents contributes to the brain or spinal cord injury associated with multiple human diseases [188]. In the animals, the release of glutamate causes dendrites and cell body swelling for around half an hour, which is preceded by intracellular organelles degeneration and pyknosis. Chemical injury mostly causes damage to neuronal tissues. The experimental glutaminergic agonist injection induces remarkable degeneration of neurons in both the spinal cord and brain [14]. This type of injury can be caused by glutamate treatment and peroxide treatment.

##### 4.8.4.1 Glutamate Treatment

Glutamate-mediated excitotoxicity is explained in the aforementioned pathophysiological portion and is crucial for neuronal cell death in different neurodegenerative disorders, including neurotrauma. Glutamate administration to neural tissue *in vitro* has been used to study neuronal injuries, including traumatic brain injury [189].

##### 4.8.4.2 Peroxide Treatment

Free radical is responsible for causing activation of secondary injury in TBI. Hydrogen peroxide administration to neural tissue stimulates oxidative damage and is used for several models of neurodegenerative diseases *in vitro*, including traumatic brain injury [189].

The major setback of this model is its specific mechanism and that it cannot mimic the common clinical human outcomes.

## 5 FUTURE PERSPECTIVE

The animal models of TBI have been established more than 40 years ago, and still, researchers are trying to develop animal models that will wholly mimic the clinical symptoms of humans. Based on the complexity of injury mechanism and cellular and molecular cascades, scientists can differentiate different types of brain injury, including focal injury, diffuse injury, and mixed injuries. Among different animal models, Marmarou’s and Shohami’s models are used for the closed head injury model, and this model reproduces diffuse and focal injury, respectively. Although, LFPI and CCI models have shown excellent reproducibility. Recently, the research on blast injury models and penetrating brain injury models has been increasing rapidly.

One of the greatest advancements has been encountered in the lateral fluid percussion model as this model can differentiate brain injury between contralateral (non-injured side) and ipsilateral (injured side) brain hemispheres and is able to differentiate diffuse and focal injury as well. The LFPI model can produce cortical and hippocampal damage that can replicate concussive injury in humans. It has now been established that LFPI and CCI models can mimic cellular and molecular cascades of human TBI. In LFPI, a higher pressure may contribute to focal injuries, whereas mild pressure may contribute to diffuse injury.

In LFPI, post-TBI complications (oxidative stress, neuroinflammation, apoptosis) are seen on the ipsilateral side. Whether post-TBI consequences are to be classified under DAI due to rapid acceleration-deceleration or not is still a doubtful matter. Effective post-TBI therapies need to be monitored against different clinical consequences in humans.

Mainly, therapeutic strategies taken in preclinical studies should have clinical relevance. Preclinical studies are needed to approach drug safety and efficacy to guide relevant clinical trials, mostly in combination therapy. Preclinically, in most cases, the treatment drugs are given after injury or before injury, which is not clinically relevant. None of the studies have been found to take full advantage of flies as this species correlates genetics with humans. Such studies could include several genetic searches that may cause an increase in sensitivity against TBI, thus providing therapeutic approaches. In addition, screening of therapeutics is going on in flies to find out effective drug treatments that may protect from post-TBI cascades.

In *in vitro* study, different injury models are required that can cover mechanical insult and stimulate multiple signaling cascades followed by injury. In the future, a type of *in vitro* study should be conducted where compounds can be tested against damage of white matter of myelinated fibre. The effect of blast injury on brain tissue is not understood well, which needs further study to find out the fundamental mechanism [190].

## CONCLUSION

Traumatic brain injury is the leading cause of death nowadays. TBI is associated with an increased risk of several neurodegenerative diseases. Mostly, TBI is observed within the age group of 1-4 years and greater than 65 years. Severe TBI is linked with intracranial injury, blood-brain barrier damage, and free radical generation. The etiology of TBI is associated with disbalance in glutamate and GABA signaling, apoptotic signaling pathway activation, and oxidative stress signaling pathway activation. TBI has a great influence on neurotransmitter levels, including serotonin, dopamine, and acetylcholine. Survived patients face complications in sexual life. There are different clinical outcomes seen after brain injury. Till now, the currently approved animal models of TBI mimic only some but not all types of human brain injury. The concept of hormesis is very important as it provides reliable data for the induction of promising therapeutic responses and plays a significant role in designing preclinical and clinical studies. For the dose-response model, hormesis, especially in the brain, is a relevant topic of interest amongst toxicologists. Mainly neurohormesis affects memory and cognitive performance, as well as antioxidants and neurodegenerative responses offered by ROS in various cellular models of TBI. However, under normal conditions, hormetic response maintains normal neurological conditions as well as optimizes neural connections, and defends neurological systems from traumatic insults [191]. Principles of hormesis described that various endogenous agonists (*e.g*., β-amyloid) as well as low doses of some stressful agents, such as toxins and some drugs, may create a hormetic response. Hormetic (biphasic) dose-response is determined quantitatively by limits in the plasticity of the neural system (as well as other biological systems). These results reveal that hormetic dose-response plays an important role in maintaining neural connection and neuroprotection. Experimental studies based on hormesis can be applied to polyphenols [192]. The studies on hormetic dose-response have recently focused attention on neuronal protection. Various *in vitro* studies have shown polyphenols to have a role in activating the heat shock proteins (Hsp) pathway, which elicits a potential role in cellular stress response by counteracting neuro-inflammatory stimuli. *In vivo* studies demonstrate that the phytochemical-rich diet has a role in enhancing neuroplasticity and neuro-inflammation stress resistance, preventing neurodegeneration [193]. Hsp family members, such as heme oxygenase-1 (HO-1), Hsp72, and Hsp70, are additionally referred to as vitagenes, and have gained much attention for their antioxidant activity. These hormetic responses are mediated *via* the activation of nuclear factor erythroid 2-related factor 2 (Nrf2) and antioxidant response elements (AREs). The hormesis principle is also applied for HO-1, and the role of HO-1 against nitrosative and oxidative stress is also a well-known topic. But the overexpression of HO-1 may accumulate its byproducts, such as carbon monoxide, iron, bilirubin, and biliverdin [194]. Recent findings show that hippocampal neurons' exposure to low levels of H_2_O_2_ activates the calcium release from the endoplasmic reticulum by opening ryanodine and IP_3_ receptors. Some shreds of evidence also show that neuronal exposure to subtoxic levels of superoxide converting to H_2_O_2_ can defend the neurons from stressors, and this neuroprotective effect potentially falls under hormesis principles. The understanding of mitochondrial ROS and hormesis is not well discovered, but the current studies show important roles of NF-kB activation in neuronal response to oxidative stress, which protects neurons from several oxidative stress by promoting SOD2 and Bcl-2 genes [195]. Preclinical models are necessary as these models let us go deeper to find mechanisms and show how these phenomena are activated after the introduction of TBI. To categorize proper diagnosis and approachable treatments, we have to create an animal model based on mild, moderate, and severe brain injury, and such types of preclinical approaches should have clinical outcomes. To achieve therapeutic interventions, the following points need to be reflected in models: establishment of the clinically relevant model; reproducibility of approved models; searching for any specific biomarkers preclinically and targeting those biomarkers. Additionally, more studies should be conducted to check the effect concerning age, sex and species on outcomes. The main problem of current treatment approaches is that most drugs (tested) cannot cross the blood-brain barrier as it hinders targeting the injured brain effectively.

## Figures and Tables

**Fig. (1):**
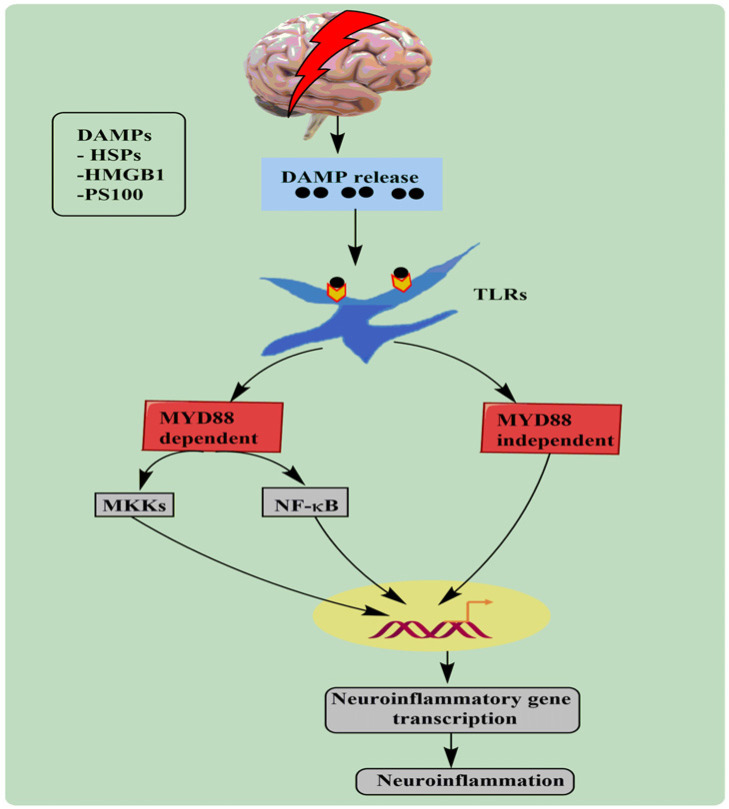
How TBI altered calcium dependent PLC pathways, oxygen species scavenging system and p53 signalling.

**Fig. (2):**
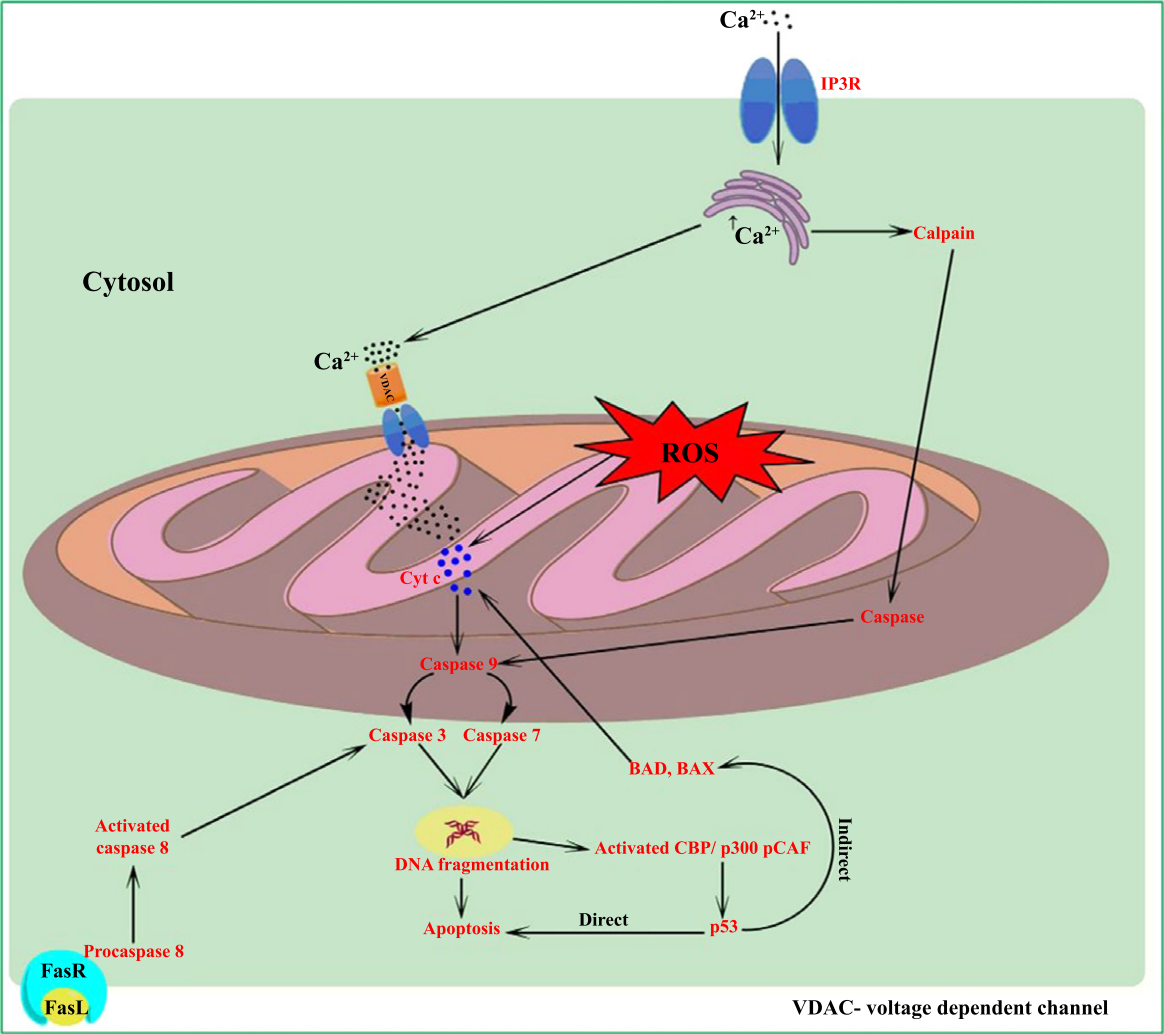
How activation of inflammatory signalling pathways leads to transcription of neuroinflammatory genes.

**Fig. (3):**
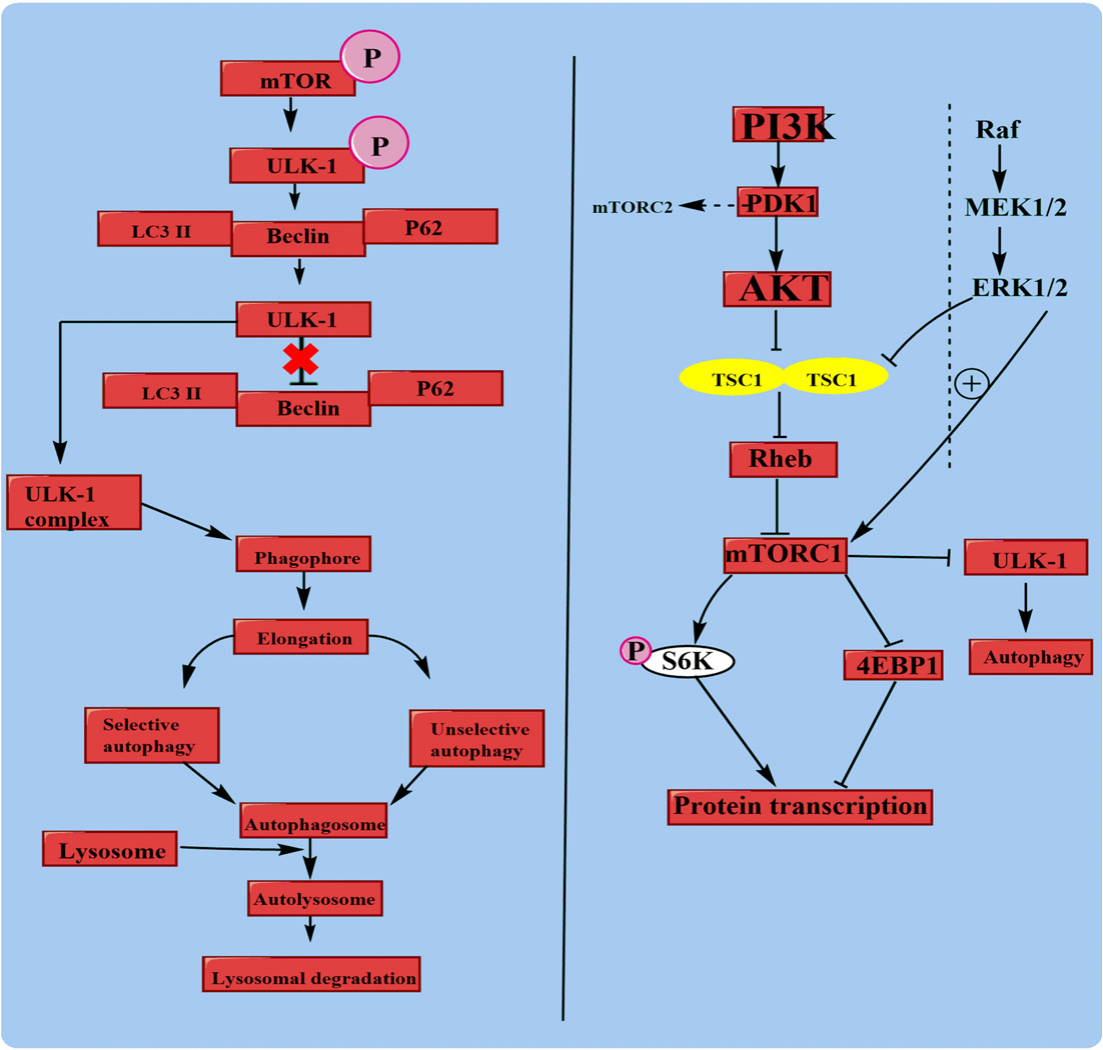
Alteration of mTOR signalling pathway post TBI.

**Table 1:** Brain area involved in different FPI.

**Model**	**Area Affected**	**Induced Pressure Range**	**Reproducibility**	**Animal Used**	**Use**	**References**
Midline FPI	Cortex, External capsule, Midbrain, Brainstem	Mild = 1.0-1.5 atm; Severe = 2.5-3.2 atm	Diffuse axonal injury	Cat; Rabbit; Rat; Dog; Sheep; Swine	Neurological evaluation	[105, 109]
Lateral FPI	Thalamus, Ipsilateral (injured area) hippocampus, Septum pellucidum, Striated septum, Amygdala	Mild = 0.9-1.5 atm; Moderate = 1.6-2.5 atm; Severe = >2.5 atm	Intracerebral haemorrhage; swelling; damage of grey matter; increased ICP; Cerebral concussion	Rat; Mouse; Swine	Memory; Cognition; Learning; Behaviour impairment	[105, 106]
Parasagittal FPI	Cortex, Hippocampus	Mild = 1.1-1.4 atm; Moderate = 1.8-2.1 atm; Severe = 2.2-2.7 atm	Increased ICP; Swelling; Neuronal loss	Rat	Post-traumatic memory and cognition deficit	[105, 107]

**Table 2:** Pressure and mortality rate of animal post blast exposure.

**Rat Position**	**Pressure (KPa)**	**Mortality Rate (%)**	**References**
Supine	103	5	[140]
	124	37	
	190	53	
Prone	103	0	
	124	11	
	190	33	
**Blast Overpressure**			
-	145	<5	[141]
	146-220	35	
	221-290	70	

**Table 3:** Methods and injury models of drosophila.

**Type of TBI**	**Method**	**Impact**	**References**
Close	High-impact trauma device	Moderate to severe	[166]
Close	Bead ruptor	Mild to severe	[167]
Close	Drosophila close head injury device	Moderate to severe	[168]

## LIST OF ABBREVIATIONS

AIF: Apoptosis-inducing Factor
AMPAR: α-Amino-3-hydroxy-5-methyl-4-isoxazole-propionic Acid Receptor
AMPK: 5' AMP-activated Protein Kinase
Apaf: Apoptotic Protease Activating Factor 1
ARE: Antioxidant Response Element
CaMK: Calcium/Calmodulin Dependent Protein Kinases
Cyt c: Cytochrome c
DAMP: Damage Associated Molecular Patterns
GABA: γ-Aminobutyric Acid
HMGB1: High Mobility Group Box 1
HO-1: Heme Oxygenase 1
HSP: Heat Shock Protein
LPO: Lipid Peroxidase
mTOR: Mammalian Target of Rapamycin
NMDAR: N-methyl-D-aspartate Receptor
Nrf2: Nuclear Factor Erythroid 2-related Factor 2
NSS: Neurological Severity Score
Rheb: Ras Homolog Enriched in Brain
ROS: Reactive Oxygen Species
SOD2: Superoxide Dismutase 2
TNF: Tumor Necrosis Factor
TSC: Tuberous Sclerosis Proteins
ULK: Unc-51 Like Autophagy Activating Kinase
